# Deep Sequencing Analysis of the *Ixodes ricinus* Haemocytome

**DOI:** 10.1371/journal.pntd.0003754

**Published:** 2015-05-13

**Authors:** Michalis Kotsyfakis, Petr Kopáček, Zdeněk Franta, Joao H. F. Pedra, José M. C. Ribeiro

**Affiliations:** 1 Institute of Parasitology, Biology Center of the Czech Academy of Sciences, Budweis, Czech Republic; 2 Department of Microbiology and Immunology, University of Maryland School of Medicine, Baltimore, Maryland, United States of America; 3 Vector Biology Section, Laboratory of Malaria and Vector Research, National Institute of Allergy and Infectious Diseases, National Institutes of Health, Rockville, Maryland, United States of America; University of Tennessee, UNITED STATES

## Abstract

**Background:**

*Ixodes ricinus* is the main tick vector of the microbes that cause Lyme disease and tick-borne encephalitis in Europe. Pathogens transmitted by ticks have to overcome innate immunity barriers present in tick tissues, including midgut, salivary glands epithelia and the hemocoel. Molecularly, invertebrate immunity is initiated when pathogen recognition molecules trigger serum or cellular signalling cascades leading to the production of antimicrobials, pathogen opsonization and phagocytosis. We presently aimed at identifying hemocyte transcripts from semi-engorged female *I*. *ricinus* ticks by mass sequencing a hemocyte cDNA library and annotating immune-related transcripts based on their hemocyte abundance as well as their ubiquitous distribution.

**Methodology/principal findings:**

*De novo* assembly of 926,596 pyrosequence reads plus 49,328,982 Illumina reads (148 nt length) from a hemocyte library, together with over 189 million Illumina reads from salivary gland and midgut libraries, generated 15,716 extracted coding sequences (CDS); these are displayed in an annotated hyperlinked spreadsheet format. Read mapping allowed the identification and annotation of tissue-enriched transcripts. A total of 327 transcripts were found significantly over expressed in the hemocyte libraries, including those coding for scavenger receptors, antimicrobial peptides, pathogen recognition proteins, proteases and protease inhibitors. Vitellogenin and lipid metabolism transcription enrichment suggests fat body components. We additionally annotated ubiquitously distributed transcripts associated with immune function, including immune-associated signal transduction proteins and transcription factors, including the STAT transcription factor.

**Conclusions/significance:**

This is the first systems biology approach to describe the genes expressed in the haemocytes of this neglected disease vector. A total of 2,860 coding sequences were deposited to GenBank, increasing to 27,547 the number so far deposited by our previous transcriptome studies that serves as a discovery platform for studies with *I*. *ricinus* biochemistry and physiology.

## Author Summary

*Ixodes ricinus* transmits Lyme disease and tick-borne encephalitis in Europe. Tick-borne pathogens ingested with a blood meal persist in the tick gut and, during the next feeding stage, disseminate through the haemolymph to pass into a vertebrate host via the salivary glands. Invertebrate immunity is initiated when tick pathogen recognition molecules trigger serum or cellular signalling cascades. We aimed to identify haemocyte transcripts from semi-engorged female *I*. *ricinus* ticks by deep sequencing a haemocyte cDNA library and by annotating immune-related transcripts based on their abundance in haemocytes and their sequence divergence when compared to their arthropod homo(ortho)logues. 15,716 extracted coding sequences (CDS) were generated in total, and 327 transcripts were significantly overexpressed in haemocytes. These included those coding for scavenger receptors, antimicrobial peptides, pathogen recognition proteins, proteases, and protease inhibitors. Transcriptional enrichment of vitellogenin and lipid metabolism effectors suggests that body fat components mediate tick innate immunity. Annotation of ubiquitously distributed transcripts associated with immune function and their phylogenetic analysis provide insights into the evolution of major protein families involved in tick innate immunity.

## Introduction

Ticks are versatile blood-feeding arthropod vectors for a wide variety of pathogens including viruses, bacteria, protozoa, fungi, and nematodes [1]. Successfully transmitted pathogens must overcome physical, cellular, and humoral barriers in the tick *en route* from being acquired (by feeding on an infected host) to infecting a naïve host during next stage feeding [2, 3]. Tick-borne pathogens have evolved strategies to survive or even proliferate in the tick gut, pass through the tick haemocoel to the salivary glands, and ultimately exploit a plethora of bioactive molecules in tick saliva to assist their transmission.

Tick salivary glands and salivary components have been extensively studied over the last decade due to their critical role in modulating host haemostasis and inflammatory or immune responses. This effort has resulted in the gradual availability of a number of salivary gland transcriptomes (sialomes) from a variety of tick species by Sanger sequencing of cDNA libraries composed of hundreds to several thousand expressed sequence tags (ESTs) [4–12]. Recently, Karim et al. [13] reported over 1.5 million ESTs from the salivary glands of the Gulf Coast tick *Amblyomma maculatum* obtained by pyrosequencing using the Roche 454 platform. For *Ixodes ricinus*, the most serious arthropod disease vector in Europe due to its transmission of Lyme borreliosis pathogens and tick-borne encephalitis virus, a mixed 454 pyrosequencing and Illumina sialotranscriptome and midgut transcriptome (also known as the mialome) have recently been reported [14–16], as well as a 454-based salivary transcriptome examining differential expression between *Bartonella henselae*-infected and non-infected ticks [17].

However, there is less transcriptomic data available from other tick tissues that play a major role at the tick-pathogen interface. To our knowledge, the only published tick midgut transcriptome was from the American dog tick *Dermacentor variabilis* and comprised over 800 distinct transcripts [18]. More recently, the 454 platform was used to identify *D*. *variabilis* immune-responsive genes upon infection with a wide range of bacterial and fungal microbes including the important tick-borne pathogen *Anaplasma marginale* [19], while others have examined differential transcript expression in adult cattle tick *Rhipicephalus (Boophilus) microplus* midguts, ovaries, or whole larvae upon feeding on a *Babesia bovis*-infected host [20–22]. However, no tick haemocyte transcriptomes are available, except for an *ex vivo* study on the haemocyte-like (phagocytic) cell line Bm-86 derived from *R*. *(B*.*) microplus* embryos [23].

In order to bridge this information gap, here we focus on comprehensively characterizing the haemocyte transcriptome of *I*. *ricinus* due to the importance of this tick in transmitting human disease. We obtained almost one million raw sequences by 454 sequencing a normalised cDNA library from *I*. *ricinus* haemocytes to provide a solid basis for annotation of almost 50 million reads produced using the Illumina platform. Furthermore, the haemocyte-derived Illumina reads were analysed with respect to over 189 million reads from a combinatorial database containing sequences from *I*. *ricinus* nymph and female salivary glands and midguts [14]. This approach allowed us to identify significantly over-represented transcripts in haemocytes and identify genes that might play a role in cellular and humoral immune reactions, particularly within the tick haemocoel. This approach provides an indispensable resource for tick innate immunity research for the development of strategies to control the transmission of tick-borne pathogens.

## Material and Methods

### Ethics statement

All animal experiments were carried out in accordance with the Animal Protection Law of the Czech Republic No. 246/1992 Sb, ethics approval No. 137/2008 and protocols approved by the responsible committee of the Institute of Parasitology, Biology Center of the Czech Academy of Sciences.

### Ticks

Male and female adult or nymphal *Ixodes ricinus* ticks were collected by flagging in a forest near the town České Budějovice in the Czech Republic. Laboratory guinea pigs reared in the animal facility of the Institute of Parasitology were used for tick feeding. For haemolymph collection, females (25 females per animal) were fed in the presence of the same number of males for six days. Semi-engorged females that had not yet entered the rapid engorgement phase [24] were forcibly removed from the host and haemolymph collected into a glass capillary from the cut front leg (0.5–2μl haemolymph per tick). Salivary glands and midguts were dissected from nymphal and adult female ticks as described previously and below [14, 15].

### Tissue collection, RNA extraction and library construction

1,080 nymphs and 420 adult females and males were attached to experimental animals for feeding and subsequent salivary gland/midgut dissections. mRNA was extracted from pools of tissues dissected from female adult ticks and nymphal ticks feeding for 3 hour periods up to 24 hours (nymphs) or 36 hours (adults), providing ten samples as follows: four samples for 0–12 h and 12–24 h for nymphal salivary glands (SG) and midguts (MG), and six samples for 0-12h, 12-24h, and 24–36 h for adult SG and MG. For the haemocyte library, total RNA was isolated from the collected haemolymph and pooled from 100 semi-engorged females. Small drops of haemolymph were immediately transferred into ice-cold RA1 buffer from the NucleoSpin RNA II kit (Macherey-Nagel) for isolation of total RNA according to the manufacturer’s protocol. One microliter of collected haemolymph contained in average 5x10^4^ haemocytes as determined previously [25, 26]. For salivary gland and midgut libraries, the experimental procedures have been described previously [14, 15]. The concentration of resulting total RNA was determined by absorbance measurement at 260/280 using a Nanodrop, and the RNA homogeneity was verified by agarose gel electrophoresis. Isolated total RNA was divided into 5 μl aliquots (3.4 μg total RNA each) and ethanol precipitated overnight at -20°C. Pellets were washed twice with 0.5 ml of ice-cold 75% ethanol and stored under1 ml of 75% ethanol at -80°C before shipping for 454 and Illumina sequencing.

### Raw sequence assembly

In addition to the reads indicated above, Illumina reads derived from the salivary gland and midgut libraries of our previous work [14, 15] were assembled together with the haemocyte Illumina reads. The detailed analysis of library construction and sequencing, sequence assembly and annotation followed standard procedures as detailed in the Supplemental Methods in the S1 File.

## Results/Discussion

### General overview of the dataset

The haemocyte-derived RNA sample (HEM) was sequenced using both 454 and Illumina technologies. A total of 926,596 sequences (median size 498 nucleotides (nt)) were obtained by 454 pyrosequencing. All Illumina sequencing followed the paired-end protocol to produce read lengths of 148 nt and a total of 49,328,982 Illumina reads. In our previous work [14], we sequenced RNA from the midguts (MG) and salivary glands (SG) of nymphs and adult female ticks of the same species; this was combined with the raw sequencing data produced here to produce a total of over 319 million Illumina-derived raw sequence reads equating to over 47.5 billion nucleotides. We used the raw sequence reads from all tissues only for contig assembly and in order to increase the possibility of a given hemocytome read to be mapped to the resulting transcript contigs. Once the contig assembly was completed, then only the sequence reads coming from the haemocytome were mapped into the resulting contigs. For a detailed analysis of sequence assembly and annotation please refer to the Materials and Methods and the Supplemental Results in the S1 File. The annotated data are available for browsing as a hyperlinked Excel file (Additional file 1: http://exon.niaid.nih.gov/transcriptome/Ixric-hem/Ir-hem-S1-web.xlsx).

A total of 15,716 CDS were obtained and classified into seven major subclasses (Table A in S1 File). Putative secreted proteins accounted for 33% of the coding sequences and 45% of the total mapped reads and included unique salivary proteins and other proteins associated with digestive or immune function including, for example, serine proteases, cathepsins, metalloproteases, and protease inhibitors. Only 1.48% of the sequence reads had a known immunity function, harbouring signatures of antimicrobial peptides, pathogen recognition proteins, or thioester-containing proteins associated with immunity. Housekeeping proteins involved in diverse metabolic pathways or the cytoskeleton accounted for 50% of the CDS and 46% of mapped reads. Transposable elements (TEs) represented 2.4% of the CDS and 0.8% of the reads, in line with the numbers of TEs found in other tick sialomes. Some vertebrate and pathogen-derived (bacterial and protozoan) coding sequences were also detected that might represent artifact from host/pathogen material contaminating/being found mainly in the digestive tissue or in the haemolymph. These exogenous CDS accounted for 734 sequences (4.6% of all CDS and 3% of all reads). Finally, there were 1,332 sequences of unknown origin or function, representing 8.5% of the total CDS and 2.61% of reads (Table A in S1 File).

When CDS were sorted according to those significantly different with respect to the number of mapped reads derived from haemocytes, 327 CDS were detected at least five-fold or greater expressed in the haemocyte library (Additional file 1, worksheet “Unclassified”, column AR; and Table 1). Of these, 35 were vertebrate derived and one was pathogen derived (from *Babesia*) and most likely represented experimental contaminants; the potential origin of each of the annotated transcripts is clearly denoted in Additional File 1. Moreover, nine TE-derived, 121 housekeeping, 129 secreted class, and 11 immunity class transcript sequences were detected. This relatively low number of haemocyte-associated sequences (only 2.1% of the total CDS representing 6.8% of the total mapped reads; Table 1) may be due to the fact that innate immunity in ticks is not exclusive to haemocytes and is also found in other tissues such as the salivary glands and midgut. Interestingly, the 11 immunity class CDS overexpressed in the haemocyte library accounted for 882,222 reads and, therefore, represented 43% of all reads mapped to the immunity class (Table A in S1 File), which might signify the transcription of some specific and abundant haemocyte markers.

**Table 1:** Functional classification of putative coding sequences (CDS) at least five-fold overexpressed in *Ixodes ricinus* haemocytes than salivary glands and midguts.

Class	Number of CDS	Number of mapped reads	Reads / CDS	% CDS	% Reads
Secreted	129	2,275,554	6,959	39.45	32.04
Immunity	11	882,222	80,202	3.36	12.42
Housekeeping	121	3,873,168	32,010	37.00	54.53
Transposable elements	9	2134	237	2.75	0.03
Vertebrate sequences	35	8343	238	10.70	0.12
Pathogen/bacterial	1	75	75	0.31	0.00
Unknown	21	61018	2,906	6.42	0.86
Total	327	7,102,514		100	100
% total from Table A in S1 File	2.08	6.79			

The selective distribution of contigs between libraries was next visualised in a heat map (Fig A in S2 File) to display the Z-scores of the normalised data for 3,915 CDS with a reads per kilobase of transcript per million reads mapped (RPKM) value of 50 or more in at least one of the three tick tissues. The heat map clearly distinguishes tissue-enriched CDS clades and a haemocyte subset.

### Analysis of coding sequences overexpressed in haemocytes

Manual annotation of the 327 CDS five-fold or greater significantly expressed in haemocytes (H-set) revealed transcripts coding for known immunity-associated proteins including members of ML domain-containing peptides (11% of H-set reads), peptidases and peptidase inhibitors, and several families of putative secreted proteins of unknown function, some of which were abundantly expressed (Table B in S1 File and Worksheet “Haemocyte-overexpressed” in Additional File 1). Lipid metabolism-associated products were well represented, including enzymes associated with fatty acid anabolism, lipases, and cholesterol transport proteins, the latter accounting for 24% of H-set reads and seven CDS with RPKM ranging from 9,000 to 48,000. These transcripts might be derived from fat body cells, similar to three transcripts coding for vitellogenins that were 10- to 20-fold overexpressed in the haemocyte library (RPKM between 168 and 1,256); tick vitellogenins are known to be synthesised by the midgut, ovaries, and fat body cells [27, 28]. P450 enzymes were overexpressed in the H-set and might be involved in prostanoid metabolism [29]. An acid sphingomyelinase was abundantly transcribed, with over 30,000 reads mapped from the haemocytome library (RPKM 1,659). Sulfotransferases were also abundant and represented 19% of H-set reads. Other notable H-set transcripts were: a zinc finger transcription factor (eight-fold overexpressed); extracellular matrix components; scavenger and Toll-like receptors (six to eight-fold overexpressed); an insulin/growth factor receptor; several transporters and ion channels including a potassium voltage-gated channel (30-times overexpressed); collagen and peritrophins; and a prolyl-hydroxylase associated with post-translational modification of collagen proline residues [30].

Some transcripts were remarkably overtranscribed in haemocytes. Three ML domain CDS involved in pathogen recognition had RPKM values ranging from 9,000 to 118,000, including one that was over 100-fold overexpressed compared to the other two tick tissues. Similar increases were observed for defensin- and microplusin-type anti-microbial peptides (180-times and 146-times overrepresented, respectively). A serine protease was assembled solely from haemocyte-derived reads and was estimated to be at least 800-times overexpressed, while a zinc carboxypeptidase that might be involved in hormone conversion was overexpressed 110-fold in the haemocyte sample. Several cystatins (inhibitors of cysteine proteases) were overrepresented between 150- and 380-fold in the haemocytome library, and a cysteine-rich peptide was 355-fold overexpressed in the same library. These transcripts can be browsed and visualised in Additional File 1 (worksheet “Haemocyte-overexpressed”; see S1 File).

TE expression has been associated with transcriptional regulation [31] and could therefore be involved in regulation of gene expression in haemocytes. The nine transposons overexpressed in haemocytes were all Class I members and included Gypsy LTR (long terminal repeat) and other non-LTR transposons.

Possible vertebrate-derived transcripts included a CDS coding for a C-C motif chemokine, five of which were 65% identical to the guinea pig homologue. However, this chemokine sequence was assembled entirely from 95 Illumina reads and five 454 reads from the H library, and none were found in the remaining libraries; most other vertebrate-derived transcripts were also found in non-haemocyte libraries. Since the haemocyte library was constructed from guinea pig-fed adult ticks, the relatively low 65% identity to the guinea pig protein may indicate that this transcript was more likely of tick origin. Other putative vertebrate-derived transcripts included those coding for interleukin-1 and class I major histocompatibility antigens, suggesting leukocyte origin.

### Discovery of immune-related proteins in *I. ricinus*

The deduced CDS (see Additional File 1 worksheet) allows for the recovery of protein sequences of interest to tick innate immunity, although many of the related CDS did not show tissue specificity. Here, we focus on the description of antimicrobial peptide families (including the nomination of novel candidate antimicrobial families), polypeptides associated with pathogen recognition (lectins, ixoderins, ML domain-containing peptides, peptidoglycan recognition proteins), protease inhibitors that might be associated with the regulation of proteinase cascades or other regulatory functions, signal transduction and transcription factors associated with immune regulation, and transcripts associated with phagocytosis.

#### Antimicrobial peptides

**Fig 1:**
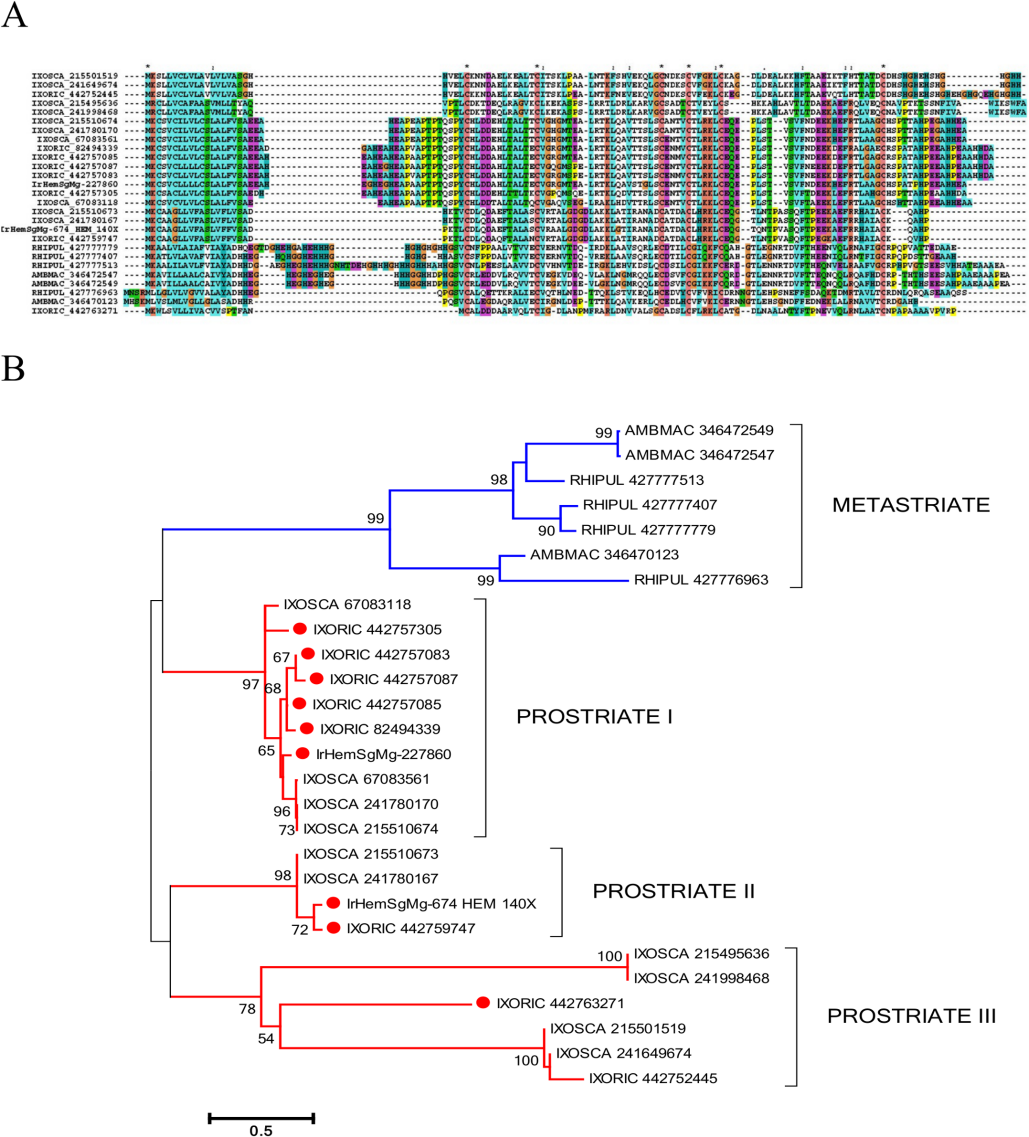
The tick microplusin family of antimicrobial peptides. A) ClustalW alignment. The symbols at the top of the figure represent (*) identity, (:) similarity, and (.) lesser similarity. B) The neighbour-joining phylogenetic tree from the alignment in (A) following 1,000 bootstraps. Sequence names are represented by the first three letters of the genus name, followed by the first three letters of the species name, followed by their GenBank gene identifier (gi) accession number. Sequences from this work start with IrHem and include IrHemSgMg-674, which is 140-times overexpressed in haemocytes. *Ixodes ricinus* sequences are indicated by a red circle. The bar at the bottom represents 50% amino acid diversity. The numbers at the nodes represent the per cent bootstrap support. Values below 50 are not shown.

Microplusin [32] is a cysteine-rich antimicrobial peptide described in the cattle tick *Rhipicephalus microplus*. One member of this family was enriched 140-times in *I. ricinus* haemocytes. Alignment of related tick sequences from GenBank (Fig 1A) with our data revealed three prostriate clades and one metastriate clade, all with multiple members (Fig 1B), indicative of a large number of genes and/or alleles in the *I. ricinus* genome. Interestingly, the *I. scapularis* and *I. ricinus* microplusins apparently lack the N-terminal histidine-rich domain important for the bacteriostatic effect of microplusin from *R. (B.) microplus* by chelating copper ions [33]. Therefore, the antibacterial effect of microplusins from *Ixodes* sp. needs experimental validation.

**Fig 2:**
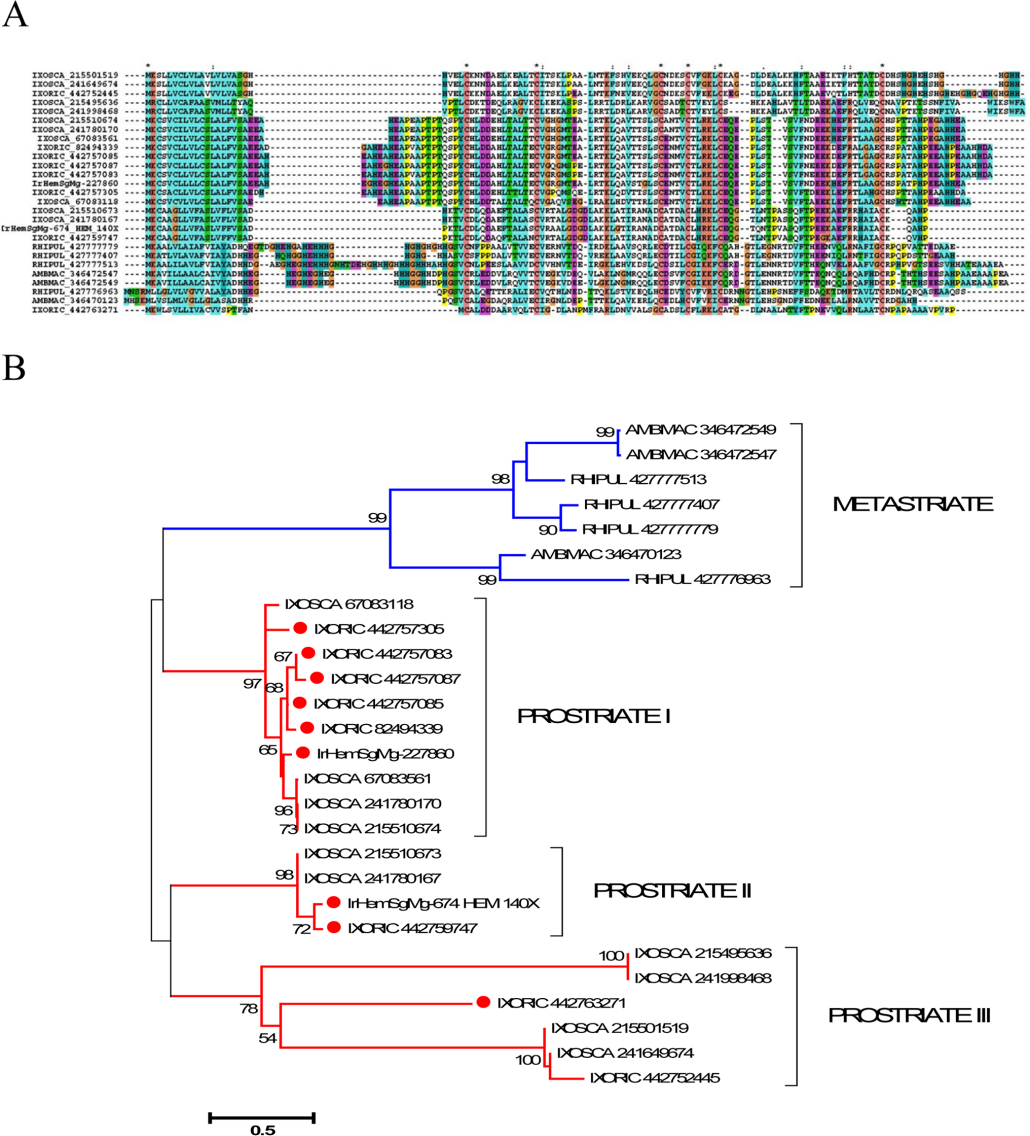
The tick defensin family of antimicrobial peptides. A) ClustalW alignment. The symbols at the top of the figure represent (*) identity and (.) lesser similarity. B) The neighbour-joining phylogenetic tree from the alignment in (A) following 1,000 bootstraps. Sequence names are represented by the first three letters of the genus name, followed by the first three letters of the species name, followed by their GenBank gene identifier (gi) accession number. *Ixodes scapularis* scapularisin and scasins are indicated. Sequences from this work start with IrHem or IrSigp. The bar at the bottom represents 20% amino acid diversity. The numbers at the nodes represent the percent bootstrap support. Values below 50 are not shown.

Defensins [34, 35] are ubiquitous antimicrobial peptides abundantly represented in ticks. In *I. ricinus*, the defensin family contained multiple genes and alleles that could be divided into two general groups with larger and smaller sequences (Fig 2A). Their phylogeny, including other tick defensins, revealed three large clades, two of which were *Ixodes* specific, while a canonical clade contained argasidae, metastriate, and prostriate sequences including scapularisins and scasins (Fig 2B). A previous analysis of the *I. scapularis* genome revealed multiple defensin-like genes in two major families: (i) scapularisins, which are structurally related to the ancient invertebrate-type defensins, and (ii) scasins, which are only distantly related to defensin-type peptides [36]. Clades I and II (Fig 2B) thus appear to be distantly related to canonical defensins and their antimicrobial activity, if any, would need to be tested.

Three tick lysozymes were identified, only one of which was relatively well expressed in haemocytes in relation to the other tissues (IrHemSgMg-127320), albeit with a low RPKM (6.0). Alignment with arthropod lysozymes (Fig B in S2 File) showed that this haemocyte lysozyme was divergent in relation to other arthropod enzymes of the same family. IrHemSgMg-62536 clustered with salivary triatomine enzymes (at 67% bootstrap support), while IrHemSgMg-119255 was a typical tick lysozyme (Fig B in S2 File).

Polypeptides containing the TIL (trypsin inhibitor-like) are known to be abundant in sialotranscriptomes, including those of male [37] and female mosquitoes [38], suggesting a potential function unrelated to blood feeding and possibly antimicrobial. Four TIL domain-containing peptides were overexpressed in the haemocyte transcriptome, suggesting a putative function in tick immunity. Phylogenetic analysis revealed the size and diversity of these peptides in ticks (Fig C in S2 File).

The 8.9 kDa protein family is uniquely found in ticks and has previously described in transcriptomes from their salivary glands [39]; however, no member of this protein family has yet been functionally characterised. The presence of two members of this family overexpressed in haemocytes supports a potential role for this family in tick immunity. Phylogenetic analysis of this family revealed a large expansion in both prostriates and metastriates (Fig D in S2 File).

Analysis of these mediators of tick innate immunity, with the exception of lysozyme, indicate large gene expansions in tick genomes compatible with an “arms race” between pathogens and their hosts. The small number of lysozymes is compatible with their role as “undertakers” of dead bacteria rather than a gene product involved in direct pathogen conflict. The sequences of the expanded gene families constitute a platform for the discovery of novel functions for family members.

#### Pathogen recognition protein families

**Fig 3:**
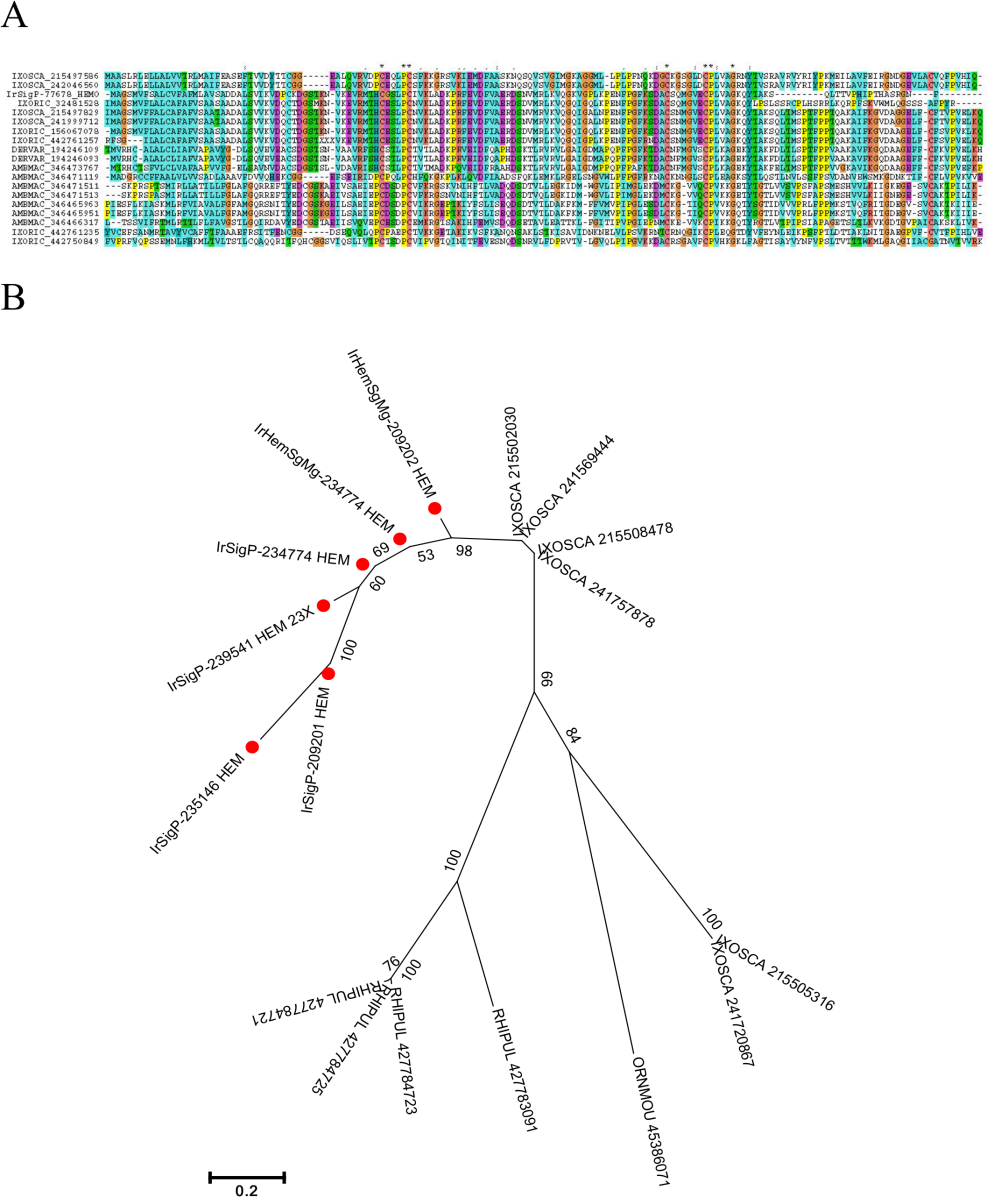
Tick ML domain proteins. A) ClustalW alignment. The symbols at the top of the figure represent (*) identity, (:) similarity, and (.) lesser similarity. B) The neighbour-joining phylogenetic tree from the alignment in (A) following 1,000 bootstraps. Sequence names are represented by the first three letters of the genus name, followed by the first three letters of the species name, followed by their GenBank gene identifier (gi) accession number. Sequences from this work start with IrHem. *Ixodes ricinus* sequences are identified with a red symbol. The bar at the bottom represents 20% amino acid diversity. The numbers at the nodes indicate the percentage bootstrap support.

The ML domain is associated with lysosomal lipid transport and pathogen recognition. Some members of this family have previously been studied in *I. ricinus* [40, 41], including a member known to be upregulated in *Borrelia* infection. Five putative coding sequences were overexpressed in haemocytes, two of which were 63- and 103-fold overexpressed. Phylogenetic analysis of tick ML-domain polypeptides (Fig 3A and 3B) indicated segregation of these proteins among species, indicative of possible gene evolution in concert with local recombination [42].

Ixoderins are tick proteins containing a PFAM fibrinogen_C domain and are implicated in immune recognition [43]. No member of this family was overexpressed in the haemocyte transcriptome, but several new members were identified. It is thus possible that the expression of members of this family is regulated post-transcriptionally. The phylogram clearly illustrates their diversity (Fig E in S2 File), which may reflect expansion of gene families associated with pathogen recognition.

IrSigP-218555 was 25-fold overexpressed in haemocytes and contains a CDDGH18_chitolectin_ chitotriosidase domain found in a large number of catalytically inactive chitinase-like lectins (chitolectins). At least six other members of this family were identified (IrSigP-237639, IrHemSgMg-233682, IrHemSgMg-233684, IrSigP-225213, IrHemSgMg-219767, IrSigP-162087). IrHemSgMg-205334, IrSigP-205335, IrHemSgMg-210952, IrSigP-226436, and IrSigP-230364 contain the smart CLECT C-type lectin (CTL) or carbohydrate-recognition domain (CRD), which may be associated with pathogen recognition. IrSigP-226436 in particular has three such domains and was 425-fold overexpressed in the midgut.

**Fig 4:**
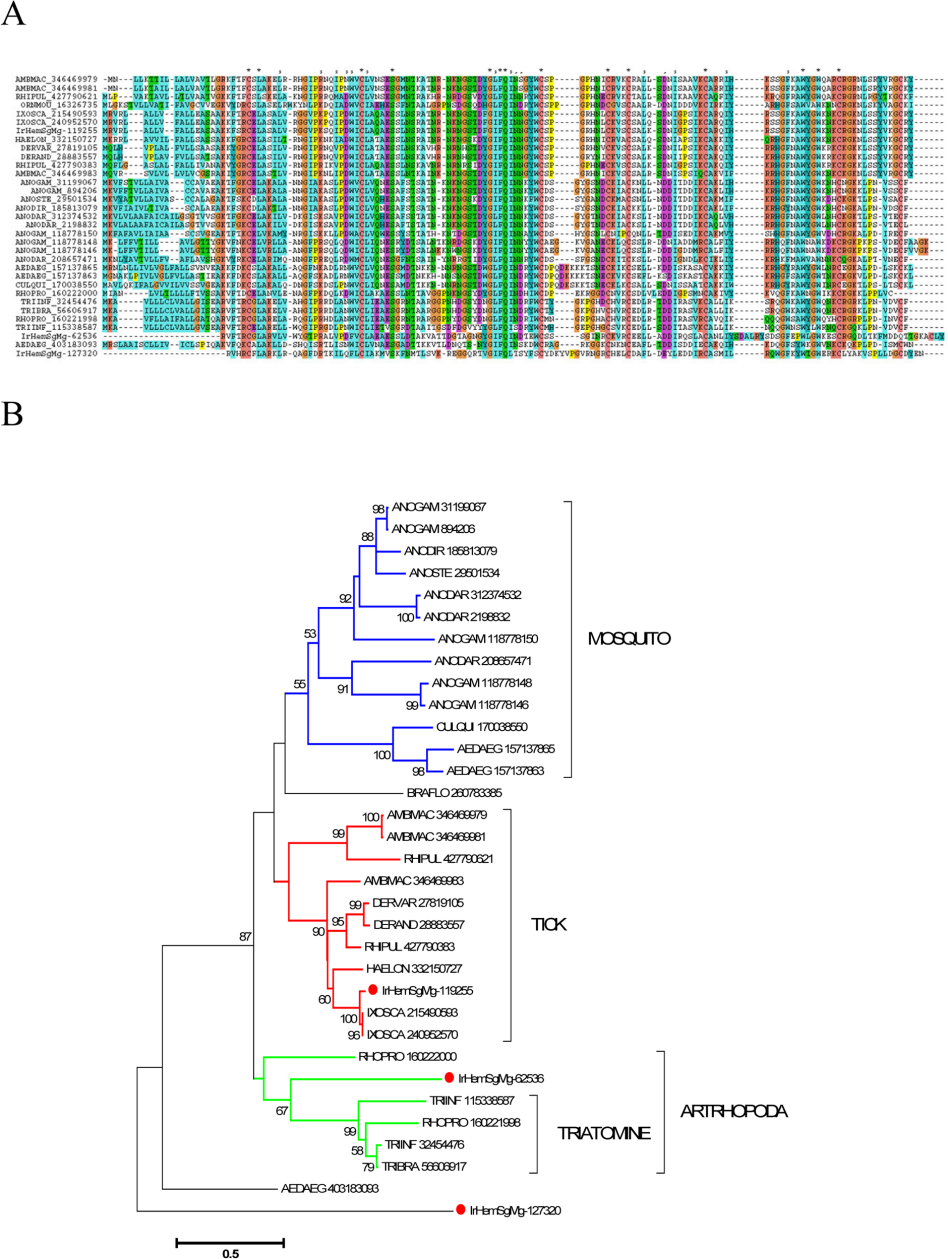
Phylogeny of invertebrate peptidoglycan recognition proteins. The A) sequence alignments and B) neighbour-joining phylogenetic tree following 1,000 bootstraps are shown. Sequence names are represented by the first three letters of the genus name, followed by the first three letters of the species name, followed by their GenBank gene identifier (gi) accession number. Sequences from this work start with IrHem or IrSigp. The bar at the bottom represents 10% amino acid diversity. The numbers at the nodes indicate the percentage bootstrap support.

Peptidoglycan recognition proteins (PGRPs) include both secreted and membrane-bound proteins that are important in the initiation of several immune cascades, including the Imd pathway in *Drosophila*; the *Drosophila* Imd protein actually has a PGRP domain [44]. Twenty-six coding sequences contained the CDD PGRP domain in this transcriptome based on comparisons with animal PGRPs homologous to Bacteriophage T3 lysozyme [45]. Alignment of these 26 protein sequences with their matches from the non-redundant protein GenBank database (Fig 4) revealed many insect-specific clades and one purely prostriate clade with *I. ricinus* and *I. scapularis* sequences, indicating a relatively large expansion of this family in *Ixodes*.

#### Proteases, protease inhibitors and reeler proteins

Proteases are associated with the innate immune system and regulate the activation of complement [46], clotting [47], and the prophenoloxidase enzyme [48]. Ticks appear to possess components of a primordial complement system [25, 49]; however, the haemolymph clotting cascade and the existence of a prophenoloxidase system have not been unequivocally demonstrated in ticks [2, 50]. IrSigP-44667 is a serine protease that was 842-times overexpressed in the haemocyte library, albeit with a relatively low RPKM of 17. Several other serine proteases were overexpressed in haemocytes, including IrHemSgMg-204852, IrHemSgMg-6004, IrSigP-233894, IrHemSgMg-195985, and IrSigP-5839, which were ten-times or more overexpressed in the haemocytome with relatively high RPKM values of between 800 and 15,921.

Protease inhibitors regulate proteases [51]. The Kunitz domain is ubiquitous in plants and animals and is associated with serine protease inhibition, and the potent salivary anti-clotting peptides found in *I. scapularis* [52, 53] are members of this family. Kunitz-containing transcripts were enriched in the haemolymph transcriptome. These might be involved in immune serine protease regulation, or, similar to the TIL domain peptides described above, it is possible that these peptides may play a role in tick immunity by targeting bacterial proteases; this is mainly indicated by the high diversity and gene expansion of the family (Fig F in S2 File).

**Fig 5:**
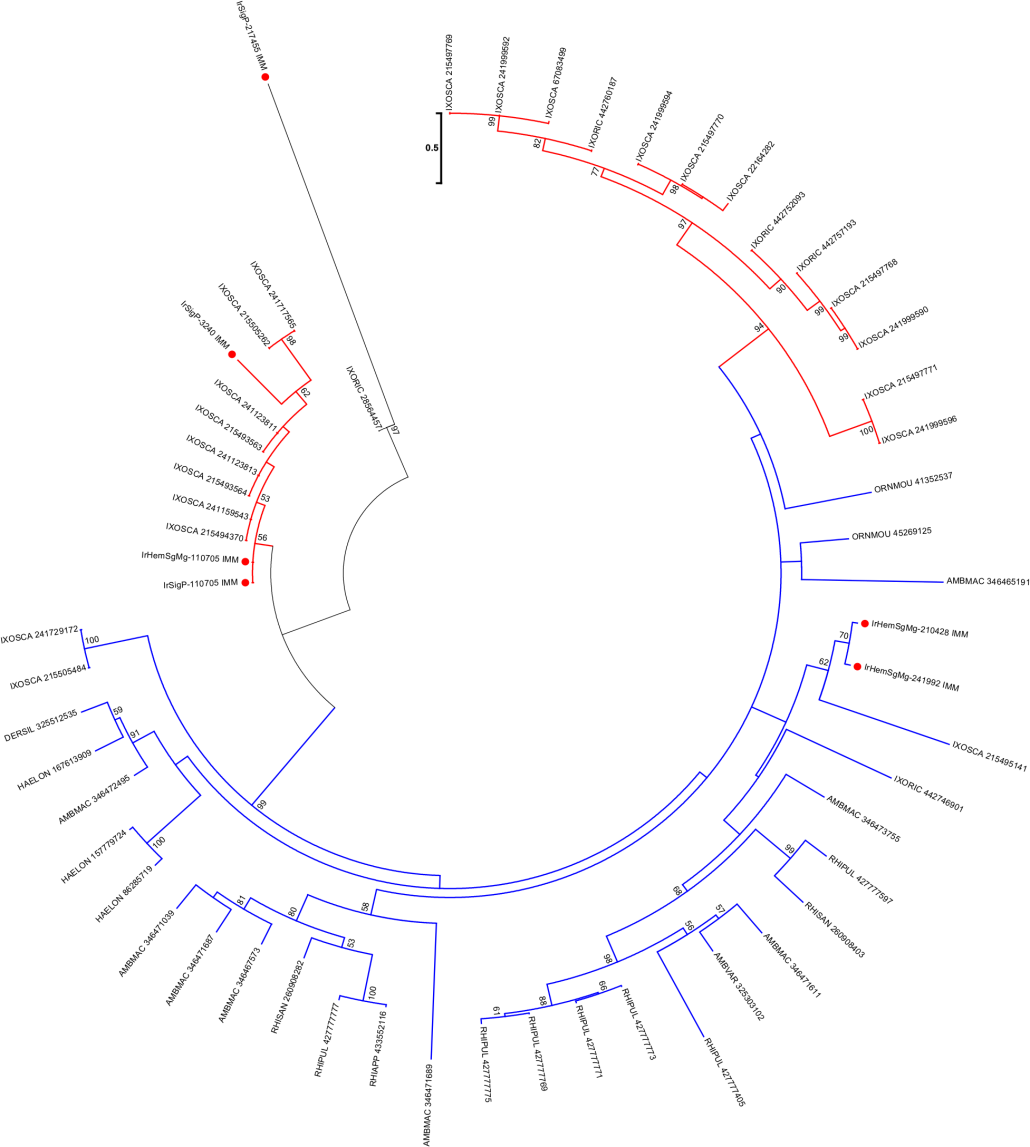
The haemocyte-enriched cystatins of *I*. *ricinus* compared to other tick cystatins. The neighbour-joining phylogenetic tree following 1,000 bootstraps is shown. Clades with significant bootstrap support are shown in colour. Sequence names are represented by the first three letters of the genus name, followed by the first three letters of the species name, followed by their GenBank gene identifier (gi) accession number. Sequences from this work start with IrHem or IrSigp and include peptide sequences highly expressed in haemocytes, identified by a red symbol. The bar at the top indicates 50% amino acid diversity. The numbers at the nodes indicate the percentage bootstrap support. Clades with strong bootstrap support are shown in colour.

Cystatins also belong to an ubiquitous protein family associated with cysteine proteinase inhibition [54]. Two salivary cystatins have been characterised in *I. scapularis* that have anti-inflammatory and immunosuppressive properties [55, 56]. Tick cystatins have also been implicated in innate immunity [57]. Since tick protein digestion is mainly accomplished by intracellular digestion with lysosomal enzymes including cysteine proteases [24], secreted haemocyte cystatins might also be directed against digestive enzyme leakage into the haemocoel. The I. ricinus transcriptome revealed several haemocyte-enriched cystatins, many of which were over 100-times overexpressed, with high RPKM’s ranging from 322 to 17,279. The phylogram of tick cystatins (Fig 5) reveals the diversity and expansion of this protein family in ticks.

**Fig 6:**
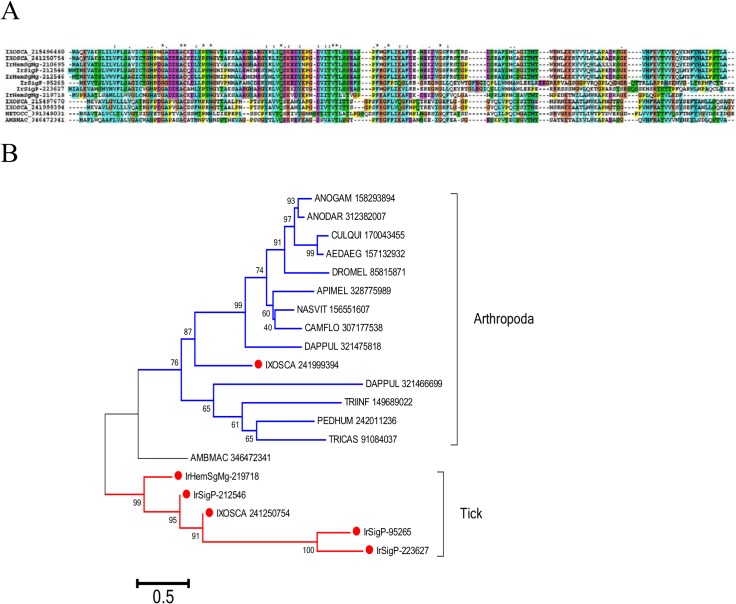
The insect defence reeler protein family of arthropods. A) ClustalW alignment. The symbols at the top of the figure represent (*) identity, (:) similarity, and (.) lesser similarity. B) The neighbour-joining phylogenetic tree from the alignment in A following 1,000 bootstraps. Sequence names are represented by the first three letters of the genus name, followed by the first three letters of the species name, followed by their GenBank gene identifier (gi) accession number. Sequences from this work start with IrHem or IrSigP. Tick sequences are identified with a red symbol. The bar at the bottom represents 50% amino acid diversity. The numbers at the nodes indicate the percentage bootstrap support.

Reeler insect defence proteins are ~160 amino acid-long secreted proteins with a single reeler domain. These proteins increase in expression upon bacterial challenge, as first demonstrated in the wild silk worm Noduler protein [58]. Knock down of this protein in *Bombyx* leads to decreased phenoloxidase activity, while injection of the recombinant protein enhances activity, suggesting that this protein is involved in the control of the melanisation cascade [59]. Several members of this family were overexpressed in the *I. ricinus* haemocytome (Fig 6A). Phylogenetic analysis of tick and other arthropod homologues reveals two distinct clades: the first has one copy per genome (marked as “Arthropoda” clade in Fig 6B), including that for *I. scapularis*, and the second is a tick-only clade that includes a second *I. scapularis* gene product and four from *I. ricinus*. Since a gene coding for a prophenoloxidase-related protein has never been identified in any tick species, the role of reeler proteins in the tick immune system will most likely differ from that reported in Lepidoptera.

#### Proteins with complement-control domains

The complement control protein (CCP) module, or Sushi repeats, is found in proteins that regulate the complement cascade. Ir-hemSgMg-216594 was over 100-fold overexpressed in haemocytes, and has an amino-terminal Sushi domain followed by a fucolectin/tachylectin motif that was previously identified in eel fucose-binding lectins [60]. IrSigP-233312 was five-fold overexpressed in haemocytes, with only a single weak match to the Sushi domain and no other recognisable domain. It has several closely related *I. scapularis* homologs but not from other species. Other proteins containing sushi domains and not overexpressed in haemocytes included IrHemSgMg-114554, which contains three sushi repeats, three complement Clr-like EGF-like domains, two tandem hyalin repeats, and two calcium-binding EGF domains. IrHemSgMg-229349, IrSigP-88055, IrHemSgMg-213204, and IrSigP-213204 contain a CDD PHA02927 domain based on secreted complement-binding proteins of viruses in addition to the CCP module. IrSigP-200536 and IrHemSgMg-200537 contain four CCP domains, one Laminin_G_3, a concanavalin A-like lectin/glucanases domain, three notch domains, and a ZnMc_pappalysin_like, zinc-dependent metalloprotease domain in its carboxy terminus, a domain associated with proteins that cleave growth factors. Proteins in this group may be associated with growth factor regulation.

#### Thioester-containing proteins

Proteins belonging to the thioester-containing protein (TEP) family are part of the vertebrate and invertebrate innate immune system and have the ability to create a thioester linkage with foreign proteins, such as in the case of the vertebrate C3 complement component and also shown in various invertebrates [2, 61]. This family was found in the *I. ricinus* transcriptome and comprised four truncated transcripts (IrSigP-194694, IrHemSgMg-194690, IrHemSgMg-194689, and IrHemSgMg-194693) coding for alpha-2-macroglobulin (A2M-2), three coding for C3-related proteins (C3-1: IrHemSgMg-234068 and IrHemSgMg-234069 and C3-3: IrSigP-232478), one insect TEP, and one macroglobulin-complement-related molecule (MCR-1, IrHemSgMg-212854) [25]. A recent study of the tissue transcription profiles of *I. ricinus* TEPs by quantitative real-time PCR revealed that A2M-2 is specifically expressed in tick haemocytes, whereas the majority of tick TEPs are expressed in tick body fat associated with tracheal trunks [62].

#### Leucine-repeat rich proteins and Toll-like receptors

**Fig 7:**
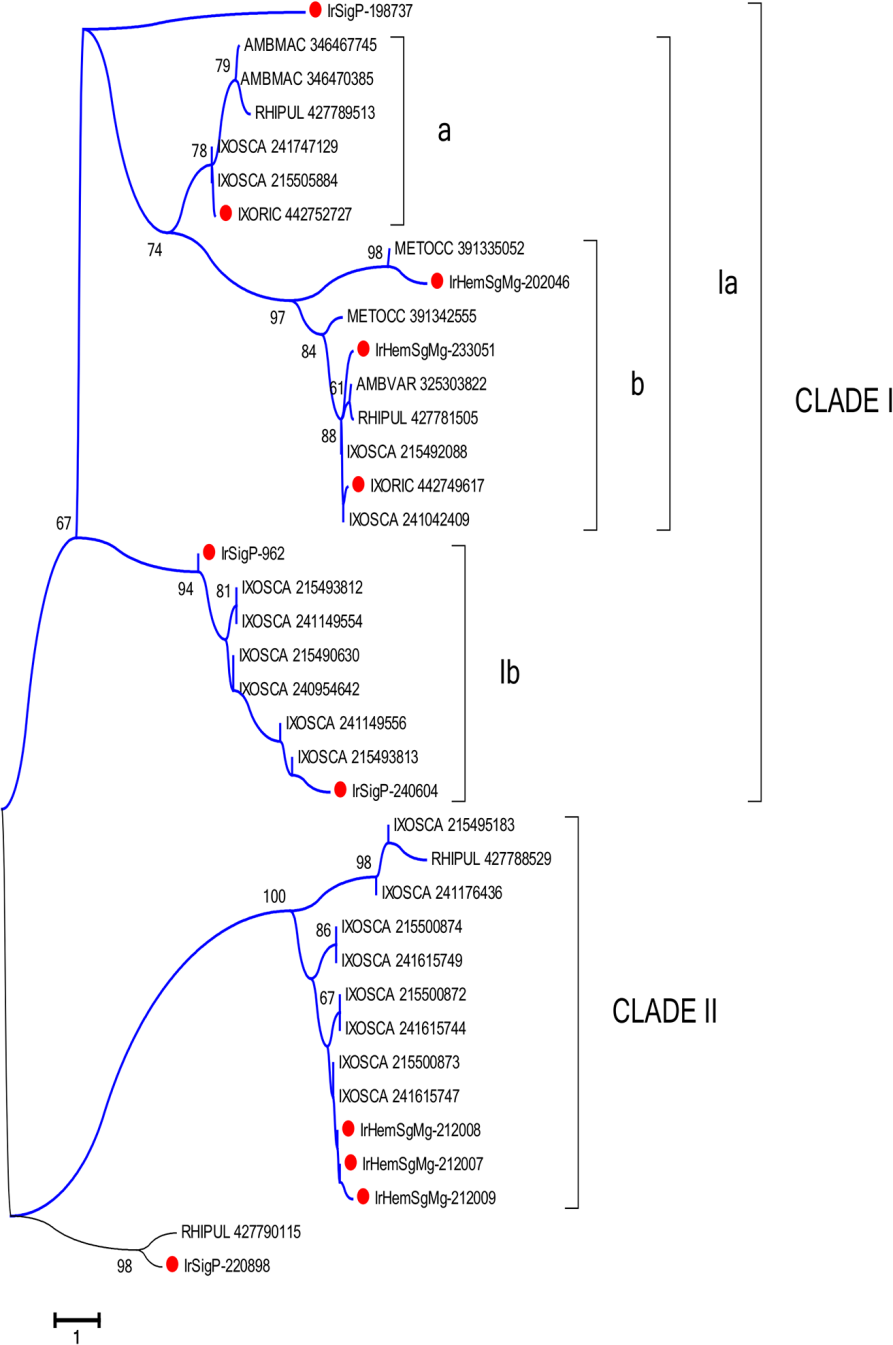
The leucine-rich repeat family of *Ixodes ricinus* compared to other tick proteins. The neighbour-joining phylogenetic tree following 1,000 bootstraps is shown. Sequence names are represented by the first three letters of the genus name, followed by the first three letters of the species name, followed by their GenBank gene identifier (gi) accession number. Sequences from this work start with IrHem or IrSigp and include peptide sequences highly expressed in haemocytes, identified by a red symbol. The bar at the top indicates 100% amino acid diversity. The numbers at the nodes indicate the percentage bootstrap support.

Membrane proteins with leucine-rich repeats are often associated with innate immunity [63, 64]. The proteins coded by IrSigP-196912, IrSigP-212008, IrHemSgMg-196912, IrSigP-198730, IrHemSgMg-1450, IrHemSgMg-196140, IrHemSgMg-209017, IrHemSgMg-200229, IrHemSgMg-83163, IrHemSgMg-74848, IrHemSgMg-117185, and IrSigP-776 are all members of a superfamily that includes Toll-like receptors and other immune regulatory proteins. The complexity of this family in ticks is illustrated by the number of clades in phylogenetic analysis, including several strong metastriate and prostriate tick clades and, in the case of sub-clade b in Clade I, the free-living predatory acari (Fig 7).

#### Additional immunity-related signal transduction proteins

Rhomboid proteins, such as IrHemSgMg-197228, IrHemSgMg-84140, IrHemSgMg-166394, and IrSigP-197227, contain the rhomboid PFAM motif. This family contains membrane proteins related to the *Drosophila* rhomboid protein. Rhomboid promotes the cleavage of the membrane-anchored TGF-alpha-like growth factor Spitz, allowing it to activate the *Drosophila* EGF receptor. IrHemSgMg-197228 and IrSigP-197227, however, best match a *Babesia bovis* rhomboid protein and may be a pathogen contaminant. IrHemSgMg-166394 best matches vertebrate proteins and may also be a contaminant.

The Imd pathway in invertebrates is associated with activation of antimicrobial peptides and involves a complex intracellular signalling cascade [65, 66]. Although the Imd pathway in ticks seems to be incomplete [67], several transcripts related to Imd pathway components were identified: IrHemSgMg-75067, IrHemSgMg-235430, and IrHemSgMg-213424 containing the CASc CDD domain for interleukin-1 beta-converting enzyme (ICE) homologues, which are cysteine-dependent aspartate-directed proteases that mediate programmed cell death similar to the *Drosophila* protein DREDD, also associated with the Imd signalling pathway [66]. IrHemSgMg-194225 and IrHemSgMg-158049, however, may be vertebrate contaminants. IrHemSgMg-239793 contains two PFAM BIR (inhibitor of apoptosis) domains and a PFAM zf-C3HC4_3 zinc finger, similar to *Drosophila* Iap2, a protein required for sustained Imd pathway responses [68]. IrHemSgMg-204270 contains the RHD-n_Dorsal_Dif and IPT_NFkappaB CDD domains, characteristic of *Drosophila* Relish, a transcription factor controlling transcripts coding for anti-microbial peptides [69].

The JAK/STAT pathway is also associated with immune activation in invertebrates following Toll-like receptor activation. The mosquito immune response is mediated by kinase activation, STAT translocation to the nucleus [70], and subsequent TEP expression. More recently, expression of a tick AMP of the 5.3-kDa family was shown to be regulated by the JAK-STAT pathway [71]. IrHemSgMg-196050 appears to be a full-length STAT transcription factor containing the full-length STAT protein KOG domain, consisting of the STAT_bind, STAT_alpha, STAT_int, and SH2 PFAM domains.

#### Phagocytosis

Innate immunity also involves phagocytosis [72], which is highly conserved between species. Phagocytosis involves cell surface receptors such as complement-like opsonisation (TEP) and scavenger receptors, along with a family with members that possess epidermal growth factor-like repeats that serve as phagocytic receptors in *Drosophila* (including the Eater, Nimrod A, Nimrod B1, Nimrod C1, and Draper proteins). The immunoglobulin superfamily receptors of the DSCAM family show intense variability and can act both as cell-surface receptors and opsonins. These proteins contain multiple Ig-like and fibronectin type 3 domains. IrHemSgMg-215754, IrHemSgMg-215755, and IrSigP-215755 have PFAM EMI and CDD TM_EGFR-like domains typical of *Drosophila* Draper. IrSigP-207640 and IrHemSgMg-212695 contain the PFAM CD36 scavenger receptor motif, and IrHemSgMg-120593 and IrSigP-115531 contain the PFAM scavenger receptor cysteine-rich (SRCR) domain, both contigs being significantly overexpressed (seven- to eight-times) in the haemocytome. Recently, a CD36 scavenger receptor was implicated in RNA interference mechanisms in ticks [73]. IrHemSgMg-75314, IrHemSgMg-132663, IrHemSgMg-81249, IrHemSgMg-207959, IrHemSgMg-140402, IrSigP-207959, IrHemSgMg-105437, IrSigP-105437, and IrHemSgMg-97649 contained one or more fibronectin type 3 domains and one or more Ig domains and may be related to the *Drosophila* DSCAM receptors.

## Conclusions

The assembly of reads from multiple tissue libraries from *I*. *ricinus* ticks (salivary glands, midguts, and haemocytes) followed by posterior mapping of the reads to the derived CDS has enabled the identification of putative haemocyte-specific transcripts of various classes. While our previous work identified thousands of salivary gland or midgut transcripts that were significantly (10-times) overexpressed in one tissue or the other, only 327 CDS were significantly (five-fold) overexpressed in the haemocyte library. This relatively small number may be due to: a) the ubiquitous distribution of immune function in epithelial cells [74], and b) as a consequence of only using adult female ticks fed for six days. Nevertheless, we were able to identify hundreds of CDS associated with antimicrobial peptides, pathogen recognition proteins, proteases and proteinase inhibitors, immune-related signal transduction proteins, and transcription factors, including the STAT protein and scavenger receptors associated with phagocytosis. Finally, we constructed a series of phylogenetic trees that provide an evolutionary perspective to tick immunity.

The identification of overexpressed transcripts in the haemocyte library matching vertebrate proteins could be due to artefactual contamination of the haemolymph with host blood during the manipulations required for haemolymph extraction. Alternatively, these matches might represent active invasion of the haemocoel by host leukocytes, or, although rare, be derived from the tick genome via a horizontal transfer event, which is known to have occurred at least once in soft ticks [75].

It is important to note that the haemocyte library was not run concomitantly with the other tissue libraries and thus bias may be present due to differences in sequencing efficiencies between batches; these comparative results should be regarded as preliminary, and quantitative PCR should be performed to verify these results in further studies. Nevertheless, the 327 overexpressed CDS make biological sense, with many expected markers of haemocyte function detected, such as scavenger receptors and antimicrobial peptides. The additional availability of 2,860 CDS from *I*. *ricinus* derived from this work, together with our previous deposition of 24,687 CDS, provide a publicly available mining platform for biochemical and physiological studies with *I*. *ricinus* that was unimaginable only a few years ago.

### Availability of supporting data

Raw sequences were deposited on the Sequence Read Archive (SRA) from the NCBI under bioproject accession PRJNA183509. The individual run files received accession numbers SRR641298, SRR641303, SRR641305, SRR641306, SRR641307, SRR641308, SRR641309, SRR641327, SRR641328, SRR641329, SRR641330, and SRR641331. A total of 2,960 coding sequences and their translations were deposited at DDBJ/EMBL/GenBank under the accession GBIH00000000. The version described in this paper is the first version, GBIH01000000.

## Supporting Information

S1 File Supplemental results, supplemental methods, supplemental references, supplemental tables and link for additional file 1. (DOCX)

S2 File Supplemental figures and their legends. (PDF)

## References

[1] de la Fuente J, Estrada-Pena A, Venzal JM, Kocan KM, Sonenshine DE. Overview: Ticks as vectors of pathogens that cause disease in humans and animals. Front Biosci. 2008;13:6938–46. 18508706 10.2741/3200

[2] Kopacek P, Hajdusek O, Buresova V, Daffre S. Tick innate immunity. Adv Exp Med Biol. 2010;708:137–62. 21528697

[3] Hajdusek O, Sima R, Ayllon N, Jalovecka M, Perner J, de la Fuente J, et al Interaction of the tick immune system with transmitted pathogens. Front Cell Infect Microbiol. 2013;3:26 10.3389/fcimb.2013.00026 23875177 PMC3712896

[4] Valenzuela JG, Francischetti IM, Pham VM, Garfield MK, Mather TN, Ribeiro JM. Exploring the sialome of the tick *Ixodes scapularis* . J Exp Biol. 2002;205(Pt 18):2843–64. 12177149 10.1242/jeb.205.18.2843

[5] Santos IK, Valenzuela JG, Ribeiro JM, de Castro M, Costa JN, Costa AM, et al Gene discovery in *Boophilus microplus*, the cattle tick: the transcriptomes of ovaries, salivary glands, and hemocytes. Ann N Y Acad Sci. 2004;1026:242–6. 15604500 10.1196/annals.1307.037

[6] Francischetti IM, My Pham V, Mans BJ, Andersen JF, Mather TN, Lane RS, et al The transcriptome of the salivary glands of the female western black-legged tick *Ixodes pacificus* (Acari: Ixodidae). Insect Biochem Mol Biol. 2005;35(10):1142–61. 16102420 10.1016/j.ibmb.2005.05.007 PMC2887698

[7] Mans BJ, Andersen JF, Schwan TG, Ribeiro JM. Characterization of anti-hemostatic factors in the argasid, *Argas monolakensis*: Implications for the evolution of blood-feeding in the soft tick family. Insect Biochem Mol Biol. 2008;38(1):22–41. 18070663 10.1016/j.ibmb.2007.09.002 PMC4274796

[8] Francischetti IM, Mans BJ, Meng Z, Gudderra N, Veenstra TD, Pham VM, et al An insight into the sialome of the soft tick, *Ornithodorus parkeri* . Insect Biochem Mol Biol. 2008;38(1):1–21. 18070662 10.1016/j.ibmb.2007.09.009 PMC2233652

[9] Anatriello E, Ribeiro JM, de Miranda-Santos IK, Brandao LG, Anderson JM, Valenzuela JG, et al An insight into the sialotranscriptome of the brown dog tick, *Rhipicephalus sanguineus* . BMC Genomics. 2010;11:450 10.1186/1471-2164-11-450 20650005 PMC3091647

[10] Chmelar J, Oliveira CJ, Rezacova P, Francischetti IM, Kovarova Z, Pejler G, et al A tick salivary protein targets cathepsin G and chymase and inhibits host inflammation and platelet aggregation. Blood. 2011;117(2):736–44. 10.1182/blood-2010-06-293241 20940421 PMC3031492

[11] Ribeiro JM, Anderson JM, Manoukis NC, Meng Z, Francischetti IM. A further insight into the sialome of the tropical bont tick, *Amblyomma variegatum* . BMC Genomics. 2011;12:136 10.1186/1471-2164-12-136 21362191 PMC3060141

[12] Francischetti IM, Anderson JM, Manoukis N, Pham VM, Ribeiro JM. An insight into the sialotranscriptome and proteome of the coarse bontlegged tick, *Hyalomma marginatum rufipes* . J Proteomics. 2011;74(12):2892–908. 10.1016/j.jprot.2011.07.015 21851864 PMC3215792

[13] Karim S, Singh P, Ribeiro JM. A deep insight into the sialotranscriptome of the Gulf Coast tick, *Amblyomma maculatum* . PLoS ONE. 2011;6(12):e28525 10.1371/journal.pone.0028525 22216098 PMC3244413

[14] Schwarz A, Tenzer S, Hackenberg M, Erhart J, Gerhold-Ay A, Mazur J, et al. A systems level analysis reveals transcriptomic and proteomic complexity in *Ixodes ricinus* midgut and salivary glands during early attachment and feeding. Mol Cel Proteomics. 2014;In Press. 10.1074/mcp.M114.039289 PMC4188998 25048707

[15] Schwarz A, von Reumont BM, Erhart J, Chagas AC, Ribeiro JM, Kotsyfakis M. De novo *Ixodes ricinus* salivary gland transcriptome analysis using two next-generation sequencing methodologies. Faseb J. 2013;27(12):4745–56. 10.1096/fj.13-232140 23964076 PMC3834774

[16] Kotsyfakis M, Schwarz A, Erhart J, Ribeiro JM. Tissue- and time-dependent transcription in *Ixodes ricinus* salivary glands and midguts when blood feeding on the vertebrate host. Sci Rep. 2015;5:9103 10.1038/srep09103 25765539 PMC4357865

[17] Liu XY, de la Fuente J, Cote M, Galindo RC, Moutailler S, Vayssier-Taussat M, et al IrSPI, a tick serine protease inhibitor involved in tick feeding and *Bartonella henselae* infection. PLoS Negl Trop Dis. 2014;8(7):e2993 10.1371/journal.pntd.0002993 25057911 PMC4109860

[18] Anderson JM, Sonenshine DE, Valenzuela JG. Exploring the mialome of ticks: an annotated catalogue of midgut transcripts from the hard tick, *Dermacentor variabilis* (Acari: Ixodidae). BMC Genomics. 2008;9:552 10.1186/1471-2164-9-552 19021911 PMC2644717

[19] Jaworski DC, Zou Z, Bowen CJ, Wasala NB, Madden R, Wang Y, et al Pyrosequencing and characterization of immune response genes from the American dog tick, *Dermacentor variabilis* (L.). Insect Mol Biol. 2010;19(5):617–30. 10.1111/j.1365-2583.2010.01037.x 20698900 PMC9327058

[20] Heekin AM, Guerrero FD, Bendele KG, Saldivar L, Scoles GA, Dowd SE, et al The ovarian transcriptome of the cattle tick, *Rhipicephalus (Boophilus) microplus*, feeding upon a bovine host infected with *Babesia bovis* . Parasit Vectors. 2013;6:276 10.1186/1756-3305-6-276 24330595 PMC4028808

[21] Heekin AM, Guerrero FD, Bendele KG, Saldivar L, Scoles GA, Dowd SE, et al Gut transcriptome of replete adult female cattle ticks, *Rhipicephalus (Boophilus) microplus*, feeding upon a *Babesia bovis*-infected bovine host. Parasitol Res. 2013;112(9):3075–90. 10.1007/s00436-013-3482-4 23749091

[22] Heekin AM, Guerrero FD, Bendele KG, Saldivar L, Scoles GA, Gondro C, et al Analysis of *Babesia bovis* infection-induced gene expression changes in larvae from the cattle tick, *Rhipicephalus (Boophilus) microplus* . Parasit Vectors. 2012;5:162 10.1186/1756-3305-5-162 22871314 PMC3436708

[23] Esteves E, Lara FA, Lorenzini DM, Costa GH, Fukuzawa AH, Pressinotti LN, et al Cellular and molecular characterization of an embryonic cell line (BME26) from the tick *Rhipicephalus (Boophilus) microplus* . Insect Biochem Mol Biol. 2008;38(5):568–80. 10.1016/j.ibmb.2008.01.006 18405834 PMC4425564

[24] Sojka D, Franta Z, Horn M, Caffrey CR, Mares M, Kopacek P. New insights into the machinery of blood digestion by ticks. Trends Parasitol. 2013;29(6):276–85. 10.1016/j.pt.2013.04.002 23664173

[25] Buresova V, Hajdusek O, Franta Z, Loosova G, Grunclova L, Levashina EA, et al Functional genomics of tick thioester-containing proteins reveal the ancient origin of the complement system. J Innate Immun. 2011;3(6):623–30. 10.1159/000328851 21811049

[26] Buresova V, Hajdusek O, Franta Z, Sojka D, Kopacek P. IrAM-An alpha2-macroglobulin from the hard tick Ixodes ricinus: characterization and function in phagocytosis of a potential pathogen *Chryseobacterium indologenes* . Devel Comp Immunol. 2009;33(4):489–98. 18948134 10.1016/j.dci.2008.09.011

[27] Boldbaatar D, Umemiya-Shirafuji R, Liao M, Tanaka T, Xuan X, Fujisaki K. Multiple vitellogenins from the *Haemaphysalis longicornis* tick are crucial for ovarian development. J Insect Physiol. 2010;56(11):1587–98. 10.1016/j.jinsphys.2010.05.019 20576517

[28] Donohue KV, Khalil SM, Sonenshine DE, Roe RM. Heme-binding storage proteins in the Chelicerata. J Insect Physiol. 2009;55(4):287–96. 10.1016/j.jinsphys.2009.01.002 19183556

[29] Yuan D, Zou Q, Yu T, Song C, Huang S, Chen S, et al Ancestral genetic complexity of arachidonic acid metabolism in Metazoa. Biochim Biophysica Acta. 2014;1841(9):1272–84. 10.1016/j.bbalip.2014.04.009 24801744

[30] de Jong L, van der Kraan I, de Waal A. The kinetics of the hydroxylation of procollagen by prolyl 4-hydroxylase. Proposal for a processive mechanism of binding of the dimeric hydroxylating enzyme in relation to the high kcat/Km ratio and a conformational requirement for hydroxylation of-X-Pro-Gly- sequences. Biochim Biophysica Acta. 1991;1079(1):103–11. 1653613 10.1016/0167-4838(91)90030-4

[31] Kelley DR, Rinn JL. Transposable elements reveal a stem cell specific class of long noncoding RNAs. Genome Biol. 2012;13(11):R107 10.1186/gb-2012-13-11-r107 23181609 PMC3580499

[32] Fogaca AC, Lorenzini DM, Kaku LM, Esteves E, Bulet P, Daffre S. Cysteine-rich antimicrobial peptides of the cattle tick *Boophilus microplus*: isolation, structural characterization and tissue expression profile. Devel Comp Immunol. 2004;28(3):191–200. 14642886 10.1016/j.dci.2003.08.001

[33] Silva FD, Rezende CA, Rossi DC, Esteves E, Dyszy FH, Schreier S, et al Structure and mode of action of microplusin, a copper II-chelating antimicrobial peptide from the cattle tick *Rhipicephalus (Boophilus) microplus* . J Biol Chem. 2009;284(50):34735–46. 10.1074/jbc.M109.016410 19828445 PMC2787336

[34] Bulet P, Stocklin R, Menin L. Anti-microbial peptides: from invertebrates to vertebrates. Immunol Rev. 2004;198:169–84. 15199962 10.1111/j.0105-2896.2004.0124.x

[35] Ganz T. Defensins: antimicrobial peptides of innate immunity. Nat Rev Immunol. 2003;3(9):710–20. 12949495 10.1038/nri1180

[36] Wang Y, Zhu S. The defensin gene family expansion in the tick *Ixodes scapularis* . Devel Comp Immunol. 2011;35(11):1128–34. 10.1016/j.dci.2011.03.030 21540051

[37] Calvo E, Pham VM, Lombardo F, Arca B, Ribeiro JM. The sialotranscriptome of adult male *Anopheles gambiae* mosquitoes. Insect Biochem Mol Biol. 2006;36(7):570–5. 16835022 10.1016/j.ibmb.2006.04.005

[38] Arca B, Lombardo F, Valenzuela JG, Francischetti IM, Marinotti O, Coluzzi M, et al An updated catalogue of salivary gland transcripts in the adult female mosquito, *Anopheles gambiae* . J Exp Biol. 2005;208(Pt 20):3971–86. 16215223 10.1242/jeb.01849

[39] Francischetti IM, Sa-Nunes A, Mans BJ, Santos IM, Ribeiro JM. The role of saliva in tick feeding. Front Biosci. 2009;14:2051–88. 19273185 10.2741/3363 PMC2785505

[40] Horackova J, Rudenko N, Golovchenko M, Havlikova S, Grubhoffer L. IrML—a gene encoding a new member of the ML protein family from the hard tick, *Ixodes ricinus* . J Vector Ecol. 2010;35(2):410–8. 10.1111/j.1948-7134.2010.00100.x 21175949

[41] Rudenko N, Golovchenko M, Edwards MJ, Grubhoffer L. Differential expression of *Ixodes ricinus* tick genes induced by blood feeding or *Borrelia burgdorferi* infection. J Med Entomol. 2005;42(1):36–41. 15691006 10.1093/jmedent/42.1.36

[42] Nei M, Rooney AP. Concerted and birth-and-death evolution of multigene families. Annu Rev Genet. 2005;39:121–52. 16285855 10.1146/annurev.genet.39.073003.112240 PMC1464479

[43] Rego RO, Hajdusek O, Kovar V, Kopacek P, Grubhoffer L, Hypsa V. Molecular cloning and comparative analysis of fibrinogen-related proteins from the soft tick *Ornithodoros moubata* and the hard tick *Ixodes ricinus* . Insect Biochem Mol Biol. 2005;35(9):991–1004. 15979000 10.1016/j.ibmb.2005.04.001

[44] Ferrandon D, Imler JL, Hoffmann JA. Sensing infection in *Drosophila*: Toll and beyond. Semin Immunol. 2004;16(1):43–53. 14751763 10.1016/j.smim.2003.10.008

[45] Kang D, Liu G, Lundstrom A, Gelius E, Steiner H. A peptidoglycan recognition protein in innate immunity conserved from insects to humans. Proc Natl Acad Sci U S A. 1998;95(17):10078–82. 9707603 10.1073/pnas.95.17.10078 PMC21464

[46] Fujita T, Matsushita M, Endo Y. The lectin-complement pathway—its role in innate immunity and evolution. Immunol Rev. 2004;198:185–202. 15199963 10.1111/j.0105-2896.2004.0123.x

[47] Krem MM, Di Cera E. Evolution of enzyme cascades from embryonic development to blood coagulation. Trends Biochem Sci. 2002;27(2):67–74. 11852243 10.1016/s0968-0004(01)02007-2

[48] Cerenius L, Soderhall K. The prophenoloxidase-activating system in invertebrates. Immunol Rev. 2004;198:116–26. 15199959 10.1111/j.0105-2896.2004.00116.x

[49] Kopacek P, Hajdusek O, Buresova V. Tick as a model for the study of a primitive complement system. Adv Exp Med Biol. 2012;710:83–93. 10.1007/978-1-4419-5638-5_9 22127888

[50] Urbanova V, Hartmann D, Grunclova L, Sima R, Flemming T, Hajdusek O, et al IrFC—An Ixodes ricinus injury-responsive molecule related to Limulus Factor C. Devel Comp Immunol. 2014;46:439–47. 10.1016/j.dci.2014.05.016 24924263

[51] Kanost MR. Serine proteinase inhibitors in arthropod immunity. Devel Comp Immunol. 1999;23(4–5):291–301. 10426423 10.1016/s0145-305x(99)00012-9

[52] Francischetti IM, Mather TN, Ribeiro JM. Penthalaris, a novel recombinant five-Kunitz tissue factor pathway inhibitor (TFPI) from the salivary gland of the tick vector of Lyme disease, *Ixodes scapularis* . Thromb Haemost. 2004;91(5):886–98. 15116248 10.1160/TH03-11-0715

[53] Francischetti IM, Valenzuela JG, Andersen JF, Mather TN, Ribeiro JM. Ixolaris, a novel recombinant tissue factor pathway inhibitor (TFPI) from the salivary gland of the tick, *Ixodes scapularis*: identification of factor X and factor Xa as scaffolds for the inhibition of factor VIIa/tissue factor complex. Blood. 2002;99(10):3602–12. 11986214 10.1182/blood-2001-12-0237

[54] Turk V, Stoka V, Turk D. Cystatins: biochemical and structural properties, and medical relevance. Front Biosci. 2008;13:5406–20. 18508595 10.2741/3089

[55] Kotsyfakis M, Karim S, Andersen JF, Mather TN, Ribeiro JM. Selective cysteine protease inhibition contributes to blood-feeding success of the tick *Ixodes scapularis* . J Biol Chem. 2007;282(40):29256–63. 17698852 10.1074/jbc.M703143200

[56] Kotsyfakis M, Sa-Nunes A, Francischetti IM, Mather TN, Andersen JF, Ribeiro JM. Antiinflammatory and immunosuppressive activity of sialostatin L, a salivary cystatin from the tick *Ixodes scapularis* . J Biol Chem. 2006;281(36):26298–307. 16772304 10.1074/jbc.M513010200

[57] Schwarz A, Valdes JJ, Kotsyfakis M. The role of cystatins in tick physiology and blood feeding. Ticks Tick Borne Dis. 2012;3(3):117–27. 10.1016/j.ttbdis.2012.03.004 22647711 PMC3412902

[58] Gandhe AS, John SH, Nagaraju J. Noduler, a novel immune up-regulated protein mediates nodulation response in insects. J Immunol. 2007;179(10):6943–51. 17982085 10.4049/jimmunol.179.10.6943

[59] Bao YY, Xue J, Wu WJ, Wang Y, Lv ZY, Zhang CX. An immune-induced reeler protein is involved in the *Bombyx mori* melanization cascade. Insect Biochem Mol Biol. 2011;41(9):696–706. 10.1016/j.ibmb.2011.05.001 21624461

[60] Honda S, Kashiwagi M, Miyamoto K, Takei Y, Hirose S. Multiplicity, structures, and endocrine and exocrine natures of eel fucose-binding lectins. J Biol Chem. 2000;275(42):33151–7. 10924498 10.1074/jbc.M002337200

[61] Armstrong PB, Quigley JP. Alpha2-macroglobulin: an evolutionarily conserved arm of the innate immune system. Dev Comp Immunol. 1999;23(4–5):375–90. 10426429 10.1016/s0145-305x(99)00018-x

[62] Urbanova V, Sima R, Sauman I, Hajdusek O, Kopacek P. Thioester-containing proteins of the tick Ixodes ricinus: Gene expression, response to microbial challenge and their role in phagocytosis of the yeast *Candida albicans* . Devel Comp Immunol. 2014;48(1):55–64. 25224405 10.1016/j.dci.2014.09.004

[63] Nurnberger T, Brunner F, Kemmerling B, Piater L. Innate immunity in plants and animals: striking similarities and obvious differences. Immunol Rev. 2004;198:249–66. 15199967 10.1111/j.0105-2896.2004.0119.x

[64] Pancer Z, Cooper MD. The evolution of adaptive immunity. Annu Rev Immunol. 2006;24:497–518. 16551257 10.1146/annurev.immunol.24.021605.090542

[65] Myllymaki H, Valanne S, Ramet M. The *Drosophila* imd signaling pathway. J Immunol. 2014;192(8):3455–62. 10.4049/jimmunol.1303309 24706930

[66] Kleino A, Silverman N. The *Drosophila* IMD pathway in the activation of the humoral immune response. Devel Comp Immunol. 2014;42(1):25–35. 10.1016/j.dci.2013.05.014 23721820 PMC3808521

[67] Severo MS, Sakhon OS, Choy A, Stephens KD, Pedra JH. The 'ubiquitous' reality of vector immunology. Cell Microbiol. 2013;15(7):1070–8 10.1111/cmi.12128 23433059 PMC3898176

[68] Valanne S, Kleino A, Myllymaki H, Vuoristo J, Ramet M. Iap2 is required for a sustained response in the *Drosophila* Imd pathway. Devel Comp Immunol. 2007;31(10):991–1001. 17343912 10.1016/j.dci.2007.01.004

[69] Imler JL, Hoffmann JA. Signaling mechanisms in the antimicrobial host defense of *Drosophila* . Curr Opin Microbiol. 2000;3(1):16–22. 10679426 10.1016/s1369-5274(99)00045-4

[70] Barillas-Mury C, Han YS, Seeley D, Kafatos FC. *Anopheles gambiae* Ag-STAT, a new insect member of the STAT family, is activated in response to bacterial infection. Embo J. 1999;18(4):959–67. 10022838 10.1093/emboj/18.4.959 PMC1171188

[71] Liu L, Dai J, Zhao YO, Narasimhan S, Yang Y, Zhang L, et al *Ixodes scapularis* JAK-STAT pathway regulates tick antimicrobial peptides, thereby controlling the agent of human granulocytic anaplasmosis. J Infect Dis. 2012;206(8):1233–41. 22859824 10.1093/infdis/jis484 PMC3448968

[72] Stuart LM, Ezekowitz RA. Phagocytosis and comparative innate immunity: learning on the fly. Nat Rev Immunol. 2008;8(2):131–41. 10.1038/nri2240 18219310

[73] Aung KM, Boldbaatar D, Umemiya-Shirafuji R, Liao M, Xuenan X, Suzuki H, et al Scavenger receptor mediates systemic RNA interference in ticks. PLoS One. 2011;6(12):e28407 10.1371/journal.pone.0028407 22145043 PMC3228737

[74] Davis MM, Engstrom Y. Immune response in the barrier epithelia: lessons from the fruit fly *Drosophila melanogaster* . J Innate Immun. 2012;4(3):273–83. 10.1159/000332947 22237424 PMC6741545

[75] Iwanaga S, Isawa H, Yuda M. Horizontal gene transfer of a vertebrate vasodilatory hormone into ticks. Nat Commun. 2014;5:3373 10.1038/ncomms4373 24556716

