# Correction: Deep Sequencing Analysis of the *Ixodes ricinus* Haemocytome

**DOI:** 10.1371/journal.pntd.0003909

**Published:** 2015-07-17

**Authors:** Michalis Kotsyfakis, Petr Kopáček, Zdeněk Franta, Joao H. F. Pedra, José M. C. Ribeiro

Figs 2 and 4 are incorrect. The authors have provided corrected versions of each here.

**Fig 2:**
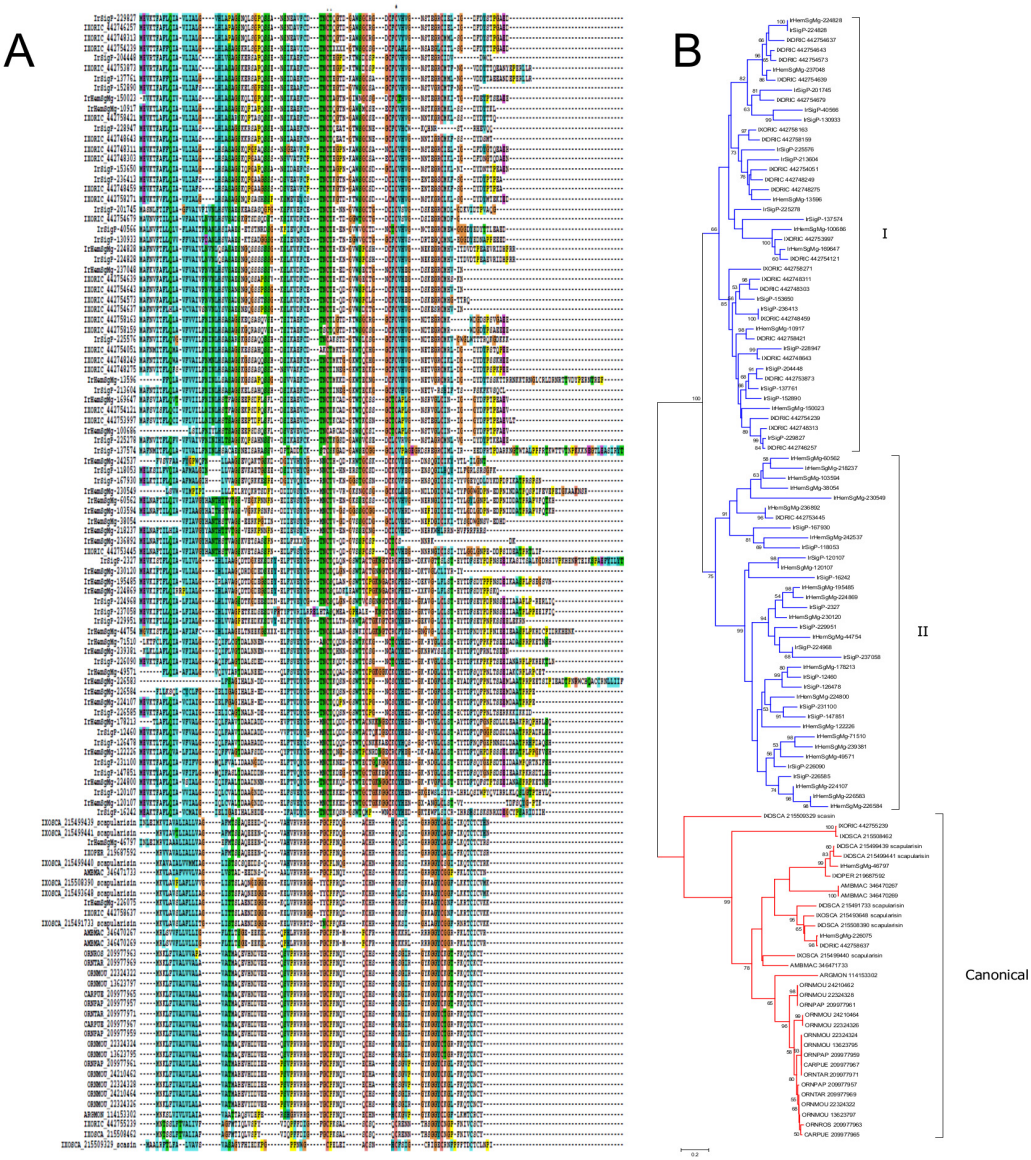
The tick defensin family of antimicrobial peptides. A) ClustalW alignment. The symbols at the top of the figure represent (*) identity and (.) lesser similarity. B) The neighbour-joining phylogenetic tree from the alignment in (A) following 1,000 bootstraps. Sequence names are represented by the first three letters of the genus name, followed by the first three letters of the species name, followed by their GenBank gene identifier (gi) accession number. *Ixodes scapularis* scapularisin and scasins are indicated. Sequences from this work start with IrHem or IrSigp. The bar at the bottom represents 20% amino acid diversity. The numbers at the nodes represent the percent bootstrap support. Values below 50 are not shown.

**Fig 4:**
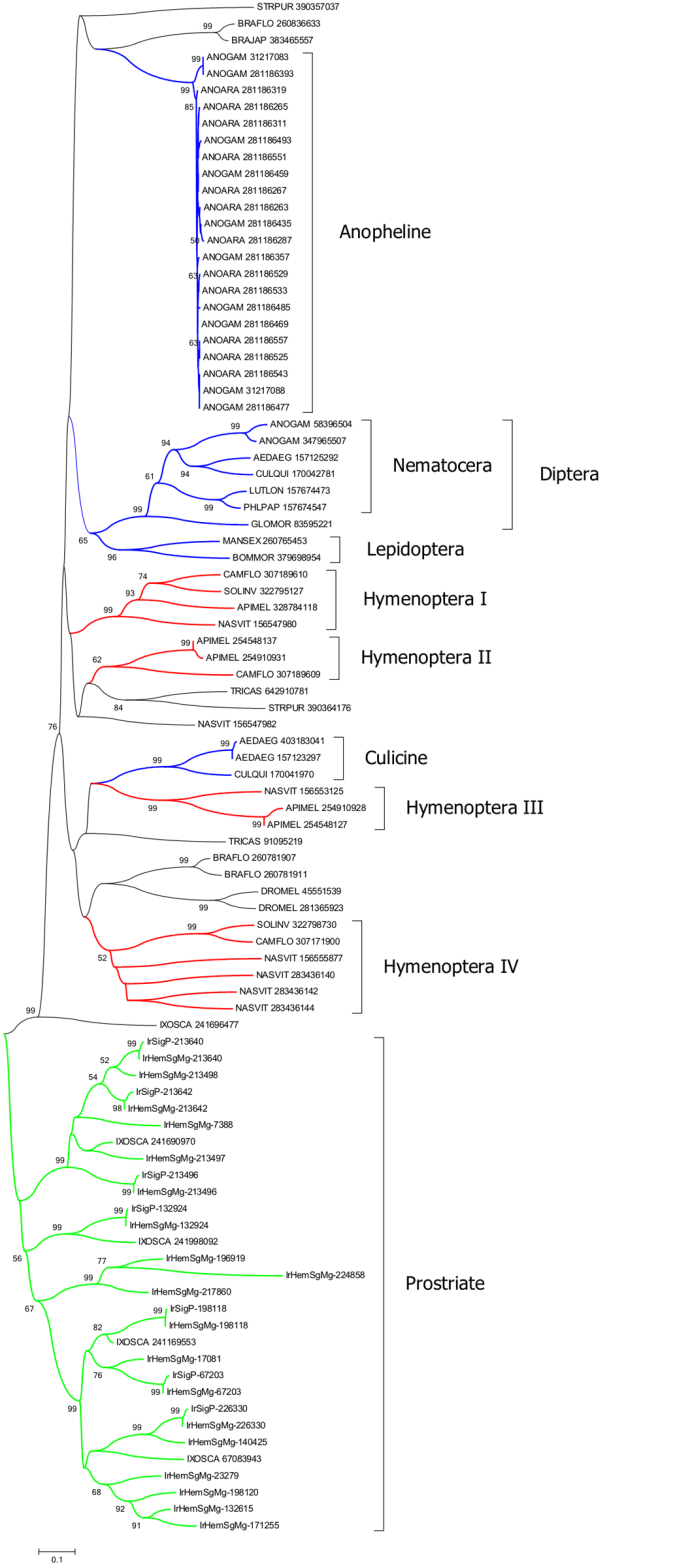
Phylogeny of invertebrate peptidoglycan recognition proteins. The neighbour-joining phylogenetic tree following 1,000 bootstraps is shown. Sequence names are represented by the first three letters of the genus name, followed by the first three letters of the species name, followed by their GenBank gene identifier (gi) accession number. Sequences from this work start with IrHem or IrSigp. The bar at the bottom represents 10% amino acid diversity. The numbers at the nodes indicate the percentage bootstrap support.
