# Supplementary material for: Deep Sequencing Analysis of the Ixodes ricinus Haemocytome
Source: PLoS Negl Trop Dis. 2015 May 13;9(5):e0003754. doi: 10.1371/journal.pntd.0003754 (PMC4430169; doi:10.1371/journal.pntd.0003754)
Supplement: S1 File — (DOCX) [file pntd.0003754.s001.docx]

**Supplemental File 1**

**Supplemental Results**

**Library construction, sequence assembly, and annotation**

The RNA Libraries of 300 bp were prepared from total RNA according the manufacturer’s instructions “TrueSeq RNA Sample Prep Guide”. According to the protocol, the total RNA is fragmented using the Covaris S2 system, first and second strand cDNA is synthesized, the overhangs are converted into blunt ends, an A-base is added to the 3' end of the blunt phosphorylated cDNA fragments, and adapters are ligated to the ends of the cDNA fragments. After PCR Enrichment of cDNA fragments, purification with AMPure beads XP, and quality control on Bioanalyzer/Qbit, the templates go on the cluster generation platform. The Illumina Cluster Station hybridised the fragments onto the flowcell and amplified them for sequencing on the Genome Analyzer IIx . Robust four-colour DNA Sequencing-By-Synthesis (SBS) technology was followed for sequencing using a 110 bp paired end protocol. The resulting fluorescent images were processed to sequences using Illumina Genome Analyzer Pipeline Analysis software v1.8.

The Illumina sequences were assembled with the Abyss [[1](#_ENREF_1)] and Trinity [[2](#_ENREF_2)] assemblers. The resulting assemblies were reassembled with the 454 sequences (after removal of vector and primer sequences) using our BLAST and CAP3 iterative method [[3](#_ENREF_3)] to generate 243,936 contigs larger than 150 nt with a median size of 880 nt. The larger open reading frames (ORFs) of contigs having a signal peptide indicative of secretion were selected to compose a putative peptide coding sequence (CDS) database. These coding sequences are prefixed with IrSigP in this work. Contigs that matched known proteins had their coding sequences extracted as follows: sequences were compared by BLASTX to several databases, including the Swissprot and Gene ontology (GO) databases, and subsets of the non-redundant protein database (NR) from the National Center for Biotechnology Information (NCBI) containing viral, bacterial, parasite, arthropods, and vertebrate sequences and all sequences from GenBank belonging to the acari and arthropoda. Contigs matching at least 70% of the length of any protein in these databases were thus extracted to the CDS database. These CDS are identified by their prefix IrHemSgMg. The combined set of 15,716 CDS with an average nucleotide length of 745 nt are reported in Additional File 1. The Illumina reads from the haemocyte, salivary gland, and midgut libraries were mapped to these CDS allowing estimation of their frequency in the different tissues. The deduced proteins from the CDS database were compared by BLASTP and RPS-BLAST to several domain databases (CDD, Pfam, Smart, and KOG [[4-7](#_ENREF_4)]). The presence of signal peptides indicative of secretion [[8](#_ENREF_8)], transmembrane domain [[9](#_ENREF_9)], mucin-type O-galactosylation [[10](#_ENREF_10)], and furin-type protein processing [[11](#_ENREF_11)] were also mapped to the protein database. The proteins were also clusterised among themselves by degrees of similarities in at least 50% of their length, helping identification of protein families.

**Supplemental Methods**

**Sequence assembly and annotation**

The raw sequence reads were assembled using the Abyss software [[1](#_ENREF_1), [12](#_ENREF_12)] with various k values (every even number from 50 to 96). Because Abyss tends to miss highly expressed contigs [[13](#_ENREF_13)], also ran the Trinity assembler [[2](#_ENREF_2)] on the raw data. Pyrosequencing data were removed from vector and primer sequences by running VecScreen. The resulting Abyss plus Trinity assemblies plus the clean pyrosequenced data were re-assembled by an iterative BLAST and CAP3 assembler [[3](#_ENREF_3)]. Coding sequences were extracted using an automated pipeline based on similarities to known proteins or by obtaining coding sequences from the larger ORF of the contigs containing a signal peptide. A non-redundant set of the coding sequences and their protein sequences were mapped into a hyperlinked Excel spreadsheet, presented as Additional File 1. Signal peptide, transmembrane domains, furin cleavage sites, and mucin-type glycosylation were determined with software from the Centre for Biological Sequence Analysis, Denmark [[8](#_ENREF_8), [9](#_ENREF_9), [11](#_ENREF_11), [14](#_ENREF_14)]. Detailed bioinformatic analysis of our pipeline can be found in our previous publication [[3](#_ENREF_3)]. To map the raw Illumina reads to the coding sequences and determine their tissue bias, raw reads from each library were BLASTed to the coding sequences using BLASTN with a word size of 25 (-W 25 switch) and allowing recovery of up to three matches. The three matches were used if they had less than two gaps and if their scores were equal to the best score. The resulting BLAST file was used to compile the number of reads each CDS received from each library and also to count the number of hits at each base of the CDS, allowing for the determination of the average CDS coverage (truncated at 30,000) and their maximum and minimum depth of coverage. To assess relative expression between haemocyte (H) and non-haemocyte (NH) tissues, a normalised ratio of the mapped reads between the two categories was performed by multiplying the number of hits derived from the H library to a normalisation number (K) and dividing this product by the number of hits derived from the NH tissues +1. One is added to the denominator to avoid eventual division by zero. These differences in the number of reads originating from different libraries by a CDS were statistically tested by a X^2^ test, the results of which were reported significant when P<0.05 and no CDS hit had an expected value of 5 or less. The RPKM (reads per thousand nucleotides per million reads) values for each sequenced library were calculated for each CDS and included in the spreadsheet as defined by the TopHat/Cufflinks program [[15](#_ENREF_15)]. Heat maps were produced with the programs gplots and heatmap.2 using R.

To assign coding sequences as being of pathogen or vertebrate origins, the top BLASTP scores of the deduced proteins against Guinea pig, vertebrate, or pathogen databases were compared to the top score derived from the BLASTP against the Acari database. If the ratio was larger than 1.25 and the e-value of the BLASTP against pathogen or vertebrate was smaller than 1e-15, then the CDS was assigned as of pathogen or vertebrate origin.

**Supplemental References**

1. Simpson JT, Wong K, Jackman SD, Schein JE, Jones SJ, Birol I. ABySS: a parallel assembler for short read sequence data. Genome Res. 2009;19(6):1117-23.

2. Grabherr MG, Haas BJ, Yassour M, Levin JZ, Thompson DA, Amit I, et al. Full-length transcriptome assembly from RNA-Seq data without a reference genome. Nat Biotechnol. 2011;29(7):644-52.

3. Karim S, Singh P, Ribeiro JM. A deep insight into the sialotranscriptome of the Gulf Coast tick, *Amblyomma maculatum*. PLoS ONE. 2011;6(12):e28525.

4. Wheeler DL, Barrett T, Benson DA, Bryant SH, Canese K, Church DM, et al. Database resources of the National Center for Biotechnology Information. Nucleic Acids Res. 2005;33(Database issue):D39-45.

5. Letunic I, Goodstadt L, Dickens NJ, Doerks T, Schultz J, Mott R, et al. Recent improvements to the SMART domain-based sequence annotation resource. Nucleic Acids Res. 2002;30(1):242-4.

6. Tatusov RL, Fedorova ND, Jackson JD, Jacobs AR, Kiryutin B, Koonin EV, et al. The COG database: an updated version includes eukaryotes. BMC Bioinformatics. 2003;4(1):41.

7. Bateman A, Birney E, Durbin R, Eddy SR, Howe KL, Sonnhammer EL. The Pfam protein families database. Nucleic Acids Res. 2000;28(1):263-6.

8. Nielsen H, Brunak S, von Heijne G. Machine learning approaches for the prediction of signal peptides and other protein sorting signals. Protein Eng. 1999;12(1):3-9.

9. Sonnhammer EL, von Heijne G, Krogh A. A hidden Markov model for predicting transmembrane helices in protein sequences. Proc Int Conf Intell Syst Mol Biol. 1998;6:175-82.

10. Hansen JE, Lund O, Tolstrup N, Gooley AA, Williams KL, Brunak S. NetOglyc: prediction of mucin type O-glycosylation sites based on sequence context and surface accessibility. Glycoconj J. 1998;15(2):115-30.

11. Duckert P, Brunak S, Blom N. Prediction of proprotein convertase cleavage sites. Protein Eng Des Sel. 2004;17(1):107-12.

12. Birol I, Jackman SD, Nielsen CB, Qian JQ, Varhol R, Stazyk G, et al. De novo transcriptome assembly with ABySS. Bioinformatics. 2009;25(21):2872-7.

13. Zhao QY, Wang Y, Kong YM, Luo D, Li X, Hao P. Optimizing de novo transcriptome assembly from short-read RNA-Seq data: a comparative study. BMC Bioinformatics. 2011;12 Suppl 14:S2.

14. Julenius K, Molgaard A, Gupta R, Brunak S. Prediction, conservation analysis, and structural characterization of mammalian mucin-type O-glycosylation sites. Glycobiology. 2005;15(2):153-64.

15. Trapnell C, Roberts A, Goff L, Pertea G, Kim D, Kelley DR, et al. Differential gene and transcript expression analysis of RNA-seq experiments with TopHat and Cufflinks. Nat Protoc. 2012;7(3):562-78.

**Supplemental Tables**

**Table A**. Functional classification of putative coding sequences (CDS) originating from salivary glands, midguts, and haemocytes of *Ixodes ricinus.*

| **Class** | **Number of CDS** | **Number of mapped reads** | **Reads / CDS** | **% CDS** | **% Reads** |
| --- | --- | --- | --- | --- | --- |
| Secreted | 5,228 | 47,633,250 | 9,111 | 33.27 | 45.52 |
| Immunity | 233 | 2,048,618 | 8,792 | 1.48 | 1.96 |
| Housekeeping | 7,805 | 48,153,655 | 6,170 | 49.66 | 46.02 |
| Transposable elements | 384 | 838,441 | 2,183 | 2.44 | 0.80 |
| Vertebrate sequences | 483 | 3,192,833 | 6,610 | 3.07 | 3.05 |
| Pathogen/bacterial | 251 | 37,013 | 147 | 1.60 | 0.04 |
| Unknown | 1,332 | 2,735,513 | 2,054 | 8.48 | 2.61 |
|  |  |  |  |  |  |
| Total | 15,716 | 104,639,323 |  | 100 | 100 |

**Table B**. Detailed functional classification of putative coding sequences (CDS) at least 5-fold overexpressed in *Ixodes ricinus* haemocytes than salivary glands and midguts

| **Class** | **Total CDS** | **Total reads** | **% Total Reads** |
| --- | --- | --- | --- |
| **Putatively related to immunity function** |  |  |  |
| Pattern recognition proteins |  |  |  |
| Chitinase-like lectin | 1 | 227 | 0.003 |
| ML domain containing peptide | 5 | 807,436 | **11.368** |
| Peptidases |  |  |  |
| Serine proteases | 14 | 256,670 | 3.614 |
| Carboxypeptidases | 2 | 1,119 | 0.016 |
| Protease inhibitors |  |  |  |
| Serpin | 1 | 316 | 0.004 |
| Kunitz domain containing protein | 9 | 73,706 | 1.038 |
| Cystatins | 8 | 265,219 | 3.734 |
| TIL domain containing protein | 4 | 1,615 | 0.023 |
| CRISP antigen 5 family | 1 | 55 | 0.001 |
| Antimicrobial peptides | 3 | 1,799 | 0.025 |
| Secreted proteins of unknown function |  |  |  |
| Family 33-19 | 7 | 80,743 | 1.137 |
| Family 37-18 | 4 | 452,841 | **6.376** |
| Family 56-12 | 6 | 1,676 | 0.024 |
| Family 82-9 | 2 | 127,898 | 1.801 |
| Family 131-6 | 2 | 32,819 | 0.462 |
| Family 165-5 | 2 | 12,331 | 0.174 |
| Family 257-4 | 2 | 2,891 | 0.041 |
| Family 6-68 | 2 | 43,257 | 0.609 |
| Family 13-46 | 2 | 4,245 | 0.060 |
| Cysteine rich | 4 | 977 | 0.014 |
| GGY rich | 1 | 412 | 0.006 |
| Methionine rich | 1 | 59 | 0.001 |
| Tryptophane rich | 5 | 10,491 | 0.148 |
| Other putative secreted proteins | 41 | 380,914 | **5.363** |
| Scavenger and Toll-like receptors | 3 | 70,135 | 0.987 |
| Other immunity-related CDS | 1 | 2,961 | 0.042 |
|  |  |  |  |
| **Housekeeping functions** |  |  |  |
| Lipid metabolism |  |  |  |
| Sphingomyelinase | 1 | 83,411 | 1.174 |
| Acyl-CoA/lipid synthase | 6 | 1,488 | 0.021 |
| Cholesterol transport protein | 7 | 1,654,381 | **23.293** |
| Lipase | 6 | 211,420 | 2.977 |
| Phosphatidylinositol transfer protein | 3 | 209 | 0.003 |
| Oxidant metabolism/Detoxification P450 enzymes | 10 | 3,568 | 0.050 |
| Carbohydrate metabolism | 2 | 63,967 | 0.901 |
| Energy metabolism | 1 | 21,480 | 0.302 |
| Signal transduction | 6 | 4,873 | 0.069 |
| Nuclear regulation | 1 | 63 | 0.001 |
| Transcription factor | 1 | 55 | 0.001 |
| Transporters and channels | 16 | 51,378 | 0.723 |
| Storage | 3 | 131,540 | 1.852 |
| Protein modification | 2 | 2,186 | 0.031 |
| Extracellular matrix | 9 | 322,946 | 4.547 |
| Mucins | 7 | 439,091 | 6.182 |
| Prolyl hydroxylase | 1 | 217 | 0.003 |
| Detoxification |  |  |  |
| Arylsulfatase | 1 | 39 | 0.001 |
| Sulfotransferase | 4 | 1,364,289 | **19.209** |
| Unknown conserved | 34 | 38,201 | 0.538 |
| Unknown conserved membrane protein | 7 | 3,330 | 0.047 |
|  |  |  |  |
| **Transposable element** | 9 | 2,134 | 0.030 |
| **Pathogen** | 1 | 75 | 0.001 |
| **Vertebrate sequences** | 35 | 8,343 | 0.117 |
| **Unknown product** | 21 | 61,018 | 0.859 |
|  |  |  |  |
| Total | 327 | 7,102,514 | 100 |

**Additional file**

Additional file 1: Hyperlinked spreadsheet with CDS annotations can be downloaded from

<http://exon.niaid.nih.gov/transcriptome/Ixric-hem/Ir-hem-S1-web.xlsx> (91 MB – web linked)
