# Supplementary material for: Deep Sequencing Analysis of the Ixodes ricinus Haemocytome
Source: PLoS Negl Trop Dis. 2015 May 13;9(5):e0003754. doi: 10.1371/journal.pntd.0003754 (PMC4430169; doi:10.1371/journal.pntd.0003754)
Supplement: S2 File — (PDF) [file pntd.0003754.s002.pdf]

Supplemental Figures

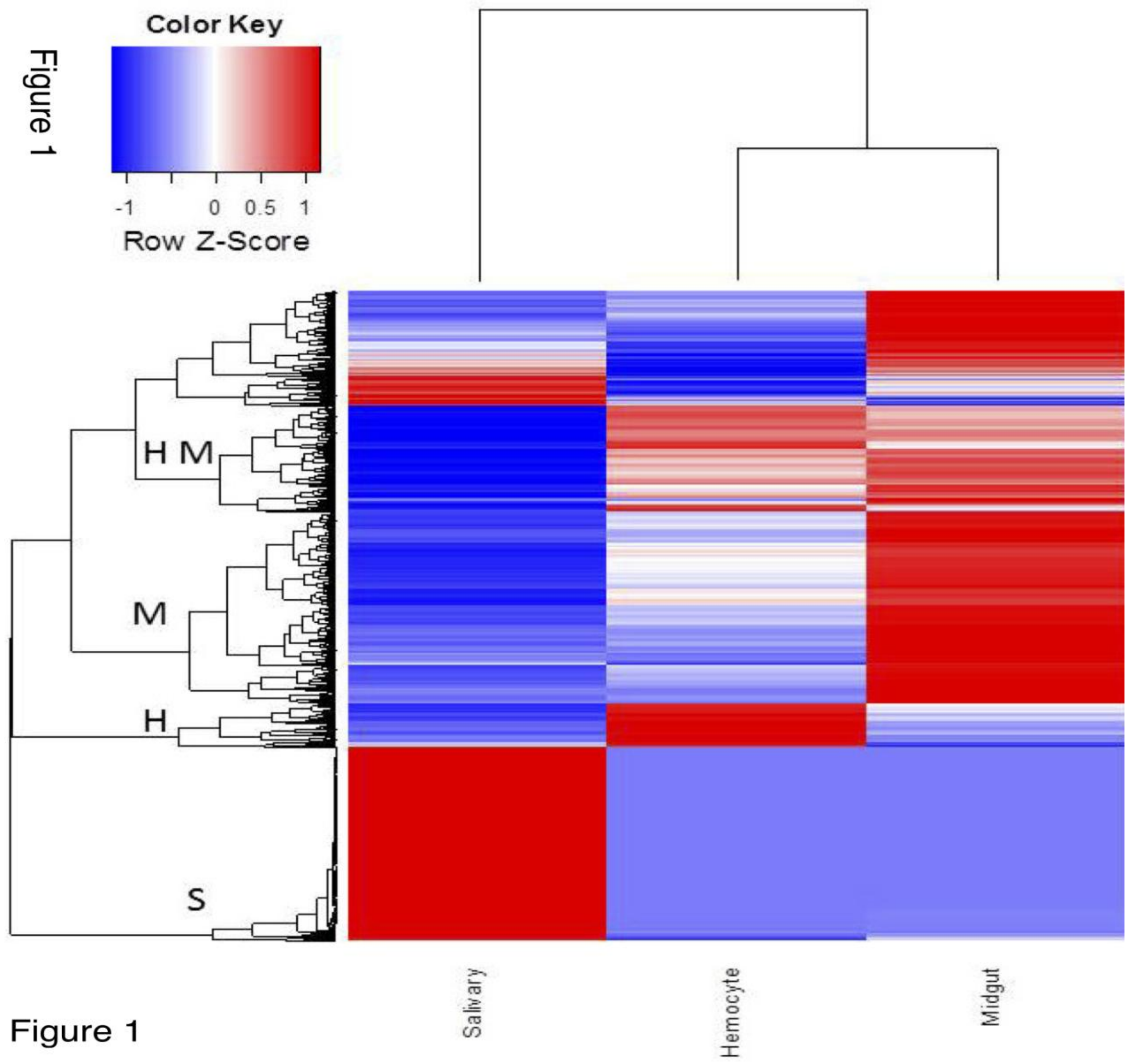

Supplemental Figure A. 3,915 CDS have a RPKM value of 50 or more in at least one of the three tick tissues. The heat map was constructed from the RPKM data for these 3,915 coding sequences according to their tissue of origin. The Z-score represents the deviation from the mean by standard deviation units of the normalised row data. The letters H, M, and S at the row cladogram nodes indicate clades that are enriched in Haemocyte, Midgut, or Salivary gland tissues, respectively.

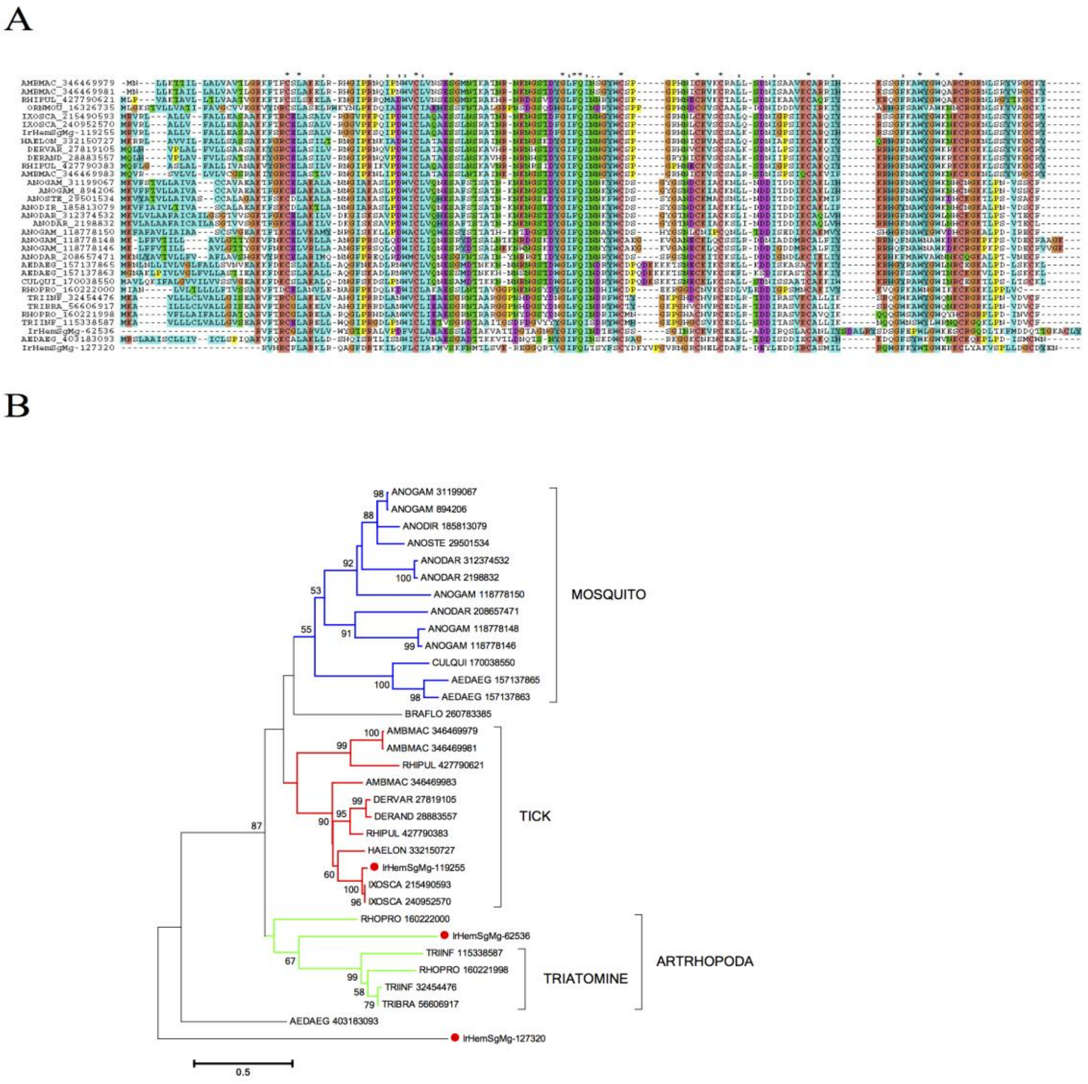

Figure 2

Supplemental Figure B. Arthropod lysozymes. A) ClustalW alignment. The symbols at the top of the figure represent (*) identity and (.) lesser similarity. B) The neighbour-joining phylogenetic tree from the alignment in (A) following 1,000 bootstraps. Sequence names are represented by the first three letters of the genus name, followed by the first three letters of the species name, followed by their GenBank gene identifier (gi) accession number. Sequences from this work start with IrHem or IrSigp and include IrHemSgMg-127320, which is expressed in haemocytes. The *Ixodes ricinus* sequences are identified with a red symbol. The bar at the bottom represents 50% amino acid diversity. The numbers at the nodes represent the per cent bootstrap support. Values below 50 are not shown.

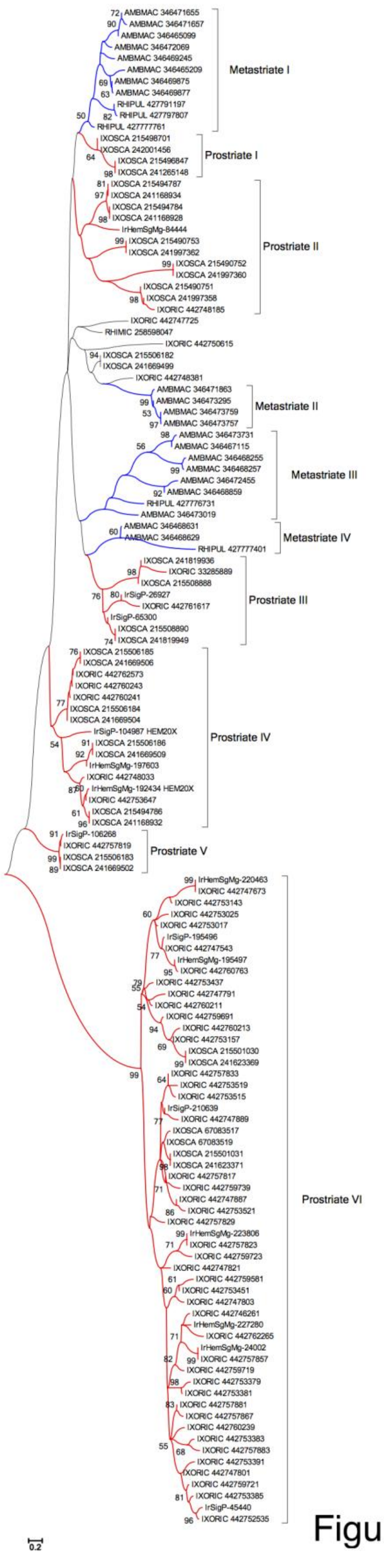

AMBMAC 346471655
AMBMAC 346471657
AMBMAC 346465099
AMBMAC 346472069
AMBMAC 346469245
AMBMAC 346465209
AMBMAC 346469875
AMBMAC 346469877
RHIPUL 427791197
RHIPUL 427797807
RHIPUL 427777761
Metastriate I
IXOSCA 215498701
IXOSCA 242001456
IXOSCA 215496847
IXOSCA 241265148
Prostriate I
IXOSCA 215494787
IXOSCA 241168934
IXOSCA 215494784
IXOSCA 241168928
IrHemSgMg-84444
IXOSCA 215490753
IXOSCA 241997362
IXOSCA 215490752
IXOSCA 241997360
IXOSCA 215490751
IXOSCA 241997358
IXORIC 442748185
Prostriate II
IXORIC 442747725
RHIMIC 258598047
IXORIC 442750615
IXOSCA 215506182
IXOSCA 241669499
IXORIC 442748381
AMBMAC 346471863
AMBMAC 346473295
AMBMAC 346473759
AMBMAC 346473757
Metastriate II
AMBMAC 346473731
AMBMAC 346467115
AMBMAC 346468255
AMBMAC 346468257
AMBMAC 346472455
AMBMAC 346468859
RHIPUL 427776731
AMBMAC 346473019
Metastriate III
AMBMAC 346468631
AMBMAC 346468629
RHIPUL 427777401
Metastriate IV
IXOSCA 241819936
IXORIC 33285889
IXOSCA 215508888
IrSigP-26927
IXORIC 442761617
IrSigP-65300
IXOSCA 215508890
IXOSCA 241819949
Prostriate III
IXOSCA 215506185
IXOSCA 241669506
IXORIC 442762573
IXORIC 442760243
IXORIC 442760241
IXOSCA 215506184
IXOSCA 241669504
IrSigP-104987 HEM20X
IXOSCA 215506186
IXOSCA 241669509
IrHemSgMg-197603
IXORIC 442748033
IrHemSgMg-192434 HEM20X
IXORIC 442753647
IXOSCA 215494786
IXOSCA 241168932
Prostriate IV
IrSigP-106268
IXORIC 442757819
IXOSCA 215506183
IXOSCA 241669502
Prostriate V
IrHemSgMg-220463
IXORIC 442747673
IXORIC 442753143
IXORIC 442753025
IXORIC 442753017
IrSigP-195496
IXORIC 442747543
IrHemSgMg-195497
IXORIC 442760763
IXORIC 442753437
IXORIC 442747791
IXORIC 442760211
IXORIC 442759691
IXORIC 442760213
IXORIC 442753157
IXOSCA 215501030
IXOSCA 241623369
IXORIC 442757833
IXORIC 442753519
IXORIC 442753515
IrSigP-210639
IXORIC 442747889
IXOSCA 67083517
IXOSCA 67083519
IXOSCA 215501031
IXOSCA 241623371
IXORIC 442757817
IXORIC 442759739
IXORIC 442747887
IXORIC 442753521
IXORIC 442757829
IrHemSgMg-223806
IXORIC 442757823
IXORIC 442759723
IXORIC 442747821
IXORIC 442759581
IXORIC 442753451
IXORIC 442747803
IXORIC 442746261
IrHemSgMg-227280
IXORIC 442762265
IrHemSgMg-24002
IXORIC 442757857
IXORIC 442759719
IXORIC 442753379
IXORIC 442753381
IXORIC 442757881
IXORIC 442757867
IXORIC 442760239
IXORIC 442753383
IXORIC 442757883
IXORIC 442753391
IXORIC 442747801
IXORIC 442759721
IXORIC 442753385
IrSigP-45440
IXORIC 442752535
Prostriate VI
0.2

Figure 3

Supplemental Figure C. The TIL domain peptide family of ticks. The neighbour-joining phylogenetic tree following 1,000 bootstraps. Sequence names are represented by the first three letters of the genus name, followed by the first three letters of the species name, followed by their GenBank gene identifier (gi) accession number. Sequences from this work start with IrHem or IrSigp and include IrSigP-201840, which is 20-fold overexpressed in haemocytes. Metastriate and prostriate clades are indicated. The bar at the bottom represents 20% amino acid diversity. The numbers at the nodes indicate the percentage bootstrap support.

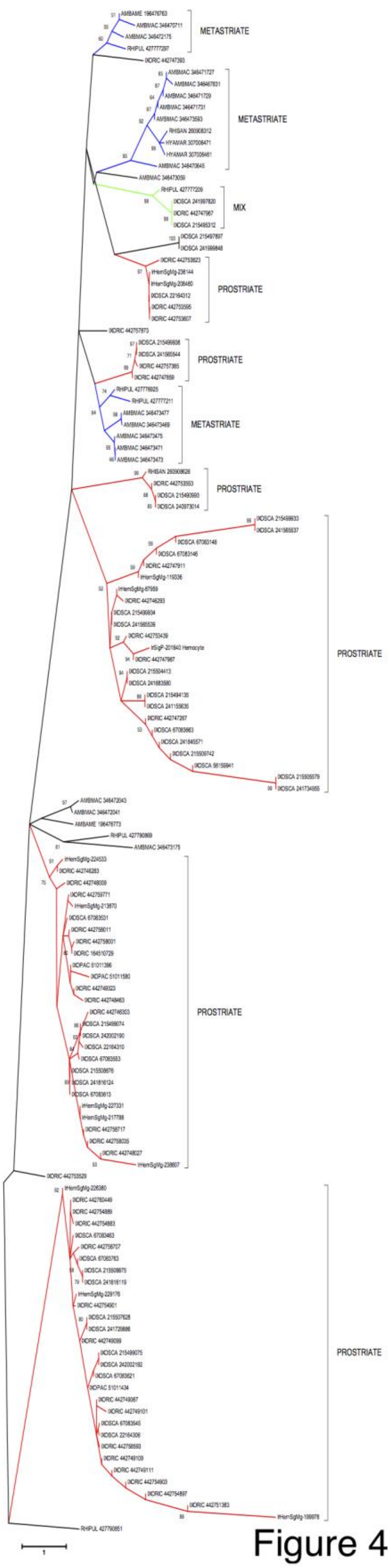
METASTRIATE
METASTRIATE
MIX
PROSTRIATE
PROSTRIATE
METASTRIATE
PROSTRIATE
PROSTRIATE
PROSTRIATE
PROSTRIATE

Figure 4

Supplemental Figure D. The 8.9 kDa family of ticks. The neighbour-joining phylogenetic tree following 1,000 bootstraps. Sequence names are represented by the first three letters of the genus name, followed by the first three letters of the species name, followed by their GenBank gene identifier (gi) accession number. Sequences from this work start with IrHem or IrSigp and include IrSigP-201840, which is 20 fold overexpressed in haemocytes. Metastriate only, prostriate only, and mixed clades are indicated. The bar at the bottom represents 100% amino acid diversity. The numbers at the nodes indicate the percentage bootstrap support.

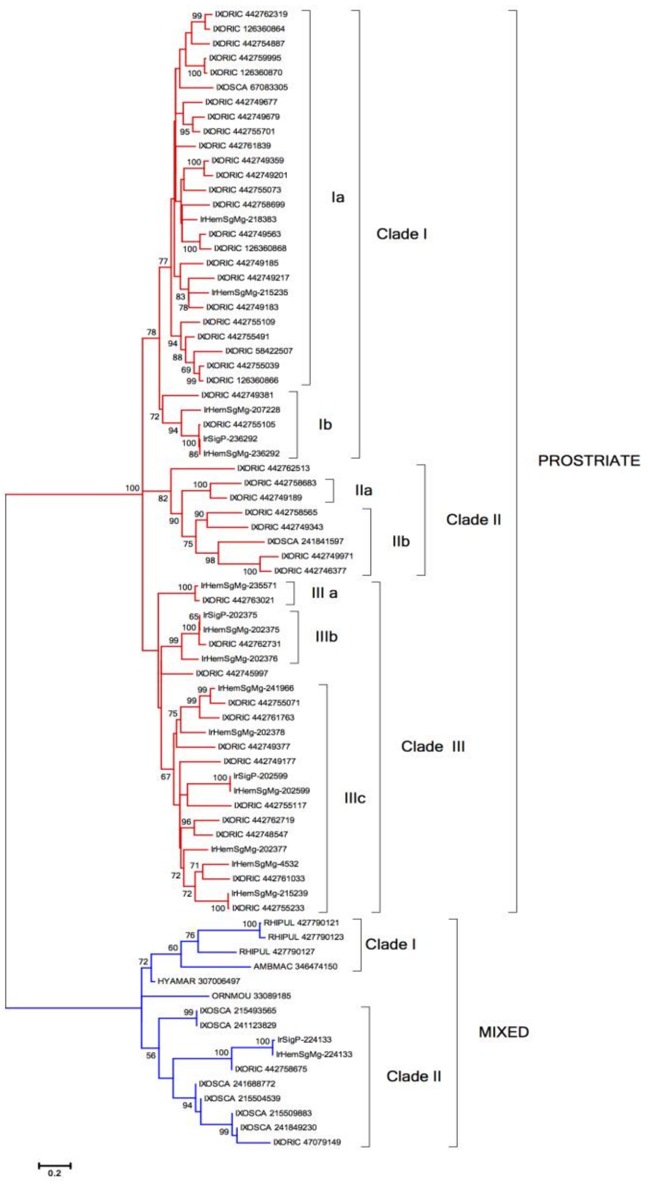

Figure 5

Supplemental Figure E. The Ixoderin family of ticks. The neighbour-joining phylogenetic tree following 1,000 bootstraps is shown. Sequence names are represented by the first three letters of the genus name, followed by the first three letters of the species name, followed by their GenBank gene identifier (gi) accession number. Sequences from this work start with IrHem or IrSigp. Clades and subclades with strong bootstrap support are indicated. The bar at the bottom represents 20% amino acid diversity. The numbers at the nodes indicate the percentage bootstrap support.

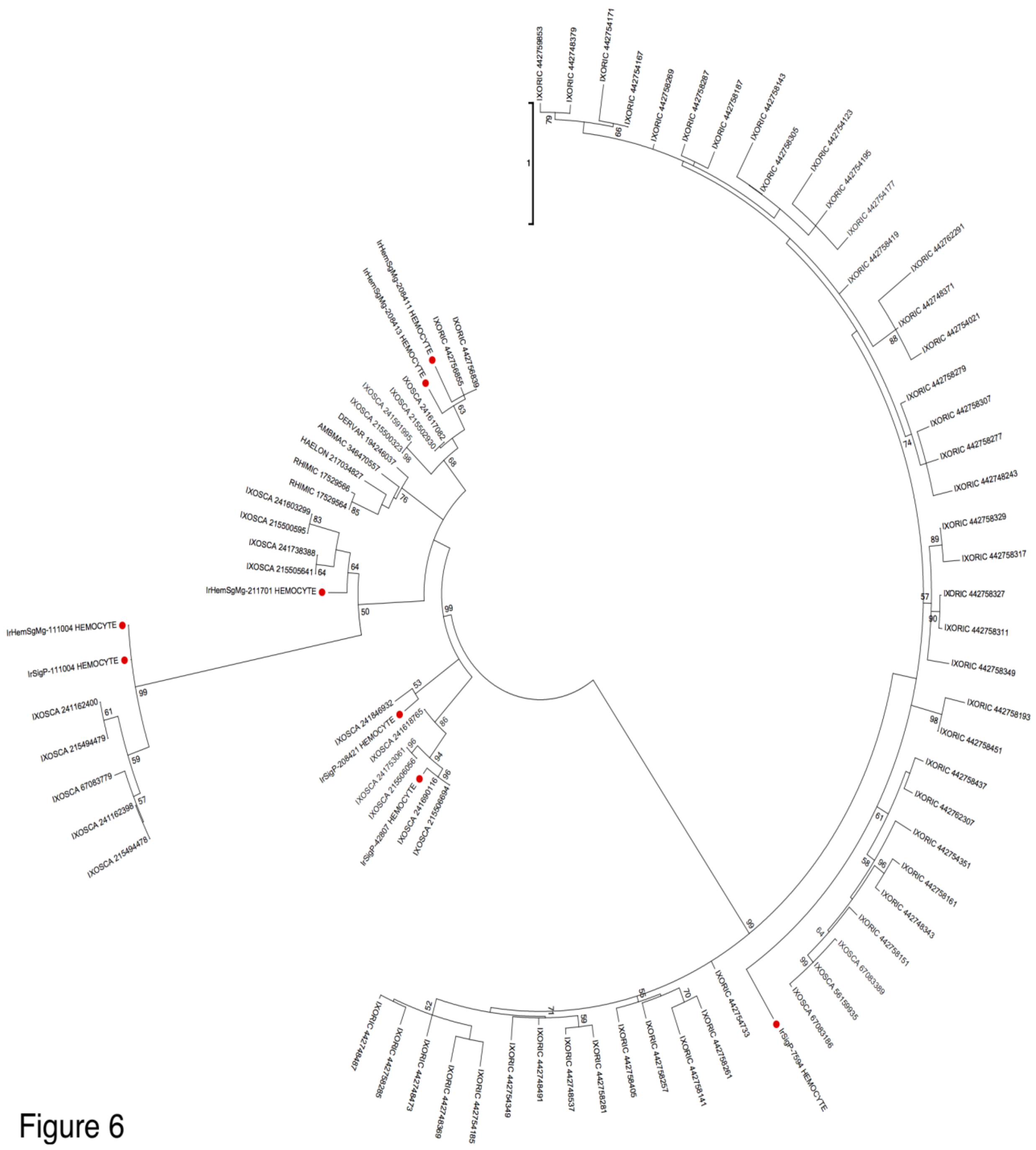

Figure 6

Supplemental Figure F. The immune Kunitz domain peptide family of *Ixodes ricinus* compared to other tick proteins. The neighbour-joining phylogenetic tree following 1,000 bootstraps is shown. Sequence names are represented by the first three letters of the genus name, followed by the first three letters of the species name, followed by their GenBank gene identifier (gi) accession number. Sequences from this work start with IrHem or IrSigp and include peptide sequences highly expressed in haemocytes, identified by a red symbol. The bar at the top indicates 100% amino acid diversity. The numbers at the nodes indicate the percentage bootstrap support.
